# Autonomic nervous system dysfunction in irritable bowel syndrome: pathophysiology and therapeutic implications

**DOI:** 10.3389/fnins.2026.1832540

**Published:** 2026-06-04

**Authors:** Yuanzhen Bai, Meilin Yu, Chunyan Weng, Jingli Xu, Mingxu Zheng, Bin Lv

**Affiliations:** 1 Department of Gastroenterology, The First Affiliated Hospital of Zhejiang Chinese Medical University (Zhejiang Provincial Hospital of Traditional Chinese Medicine), Hangzhou, China; 2 Key Laboratory of Digestive Pathophysiology of Zhejiang Province, Hangzhou, China

**Keywords:** autonomic nervous system, enteric nervous system, gut-brain axis, irritable bowel syndrome, vagus nerve stimulation

## Abstract

This review synthesizes current evidence on autonomic nervous system (ANS) dysfunction in irritable bowel syndrome (IBS). Patients with IBS often exhibit sympathovagal imbalance–reduced vagal tone with relative sympathetic hyperactivity–which correlates with symptom severity and shows subtype specificity. The ANS orchestrates bidirectional brain–gut communication via interactions with psychosocial factors, low-grade neuroinflammation, and the gut microbiota. Key mechanisms include vagal afferent signaling by microbial metabolites, sympathetic regulation of mucosal immunity, stress-induced disruption of autonomic homeostasis, and neuroplastic changes in intestinal and central pain pathways. Emerging evidence supports therapeutic targeting of autonomic circuits through vagus nerve stimulation, pharmacological modulation of serotonin and adrenergic receptors, and microbiome-based interventions. Current challenges include methodological limitations in assessing neural dynamics and insufficient integration of multi-system interactions. Future research should employ multi-omics approaches to elucidate pathway-specific mechanisms and develop precision medicine strategies for this heterogeneous disorder.

## Introduction

**FIGURE 1:**
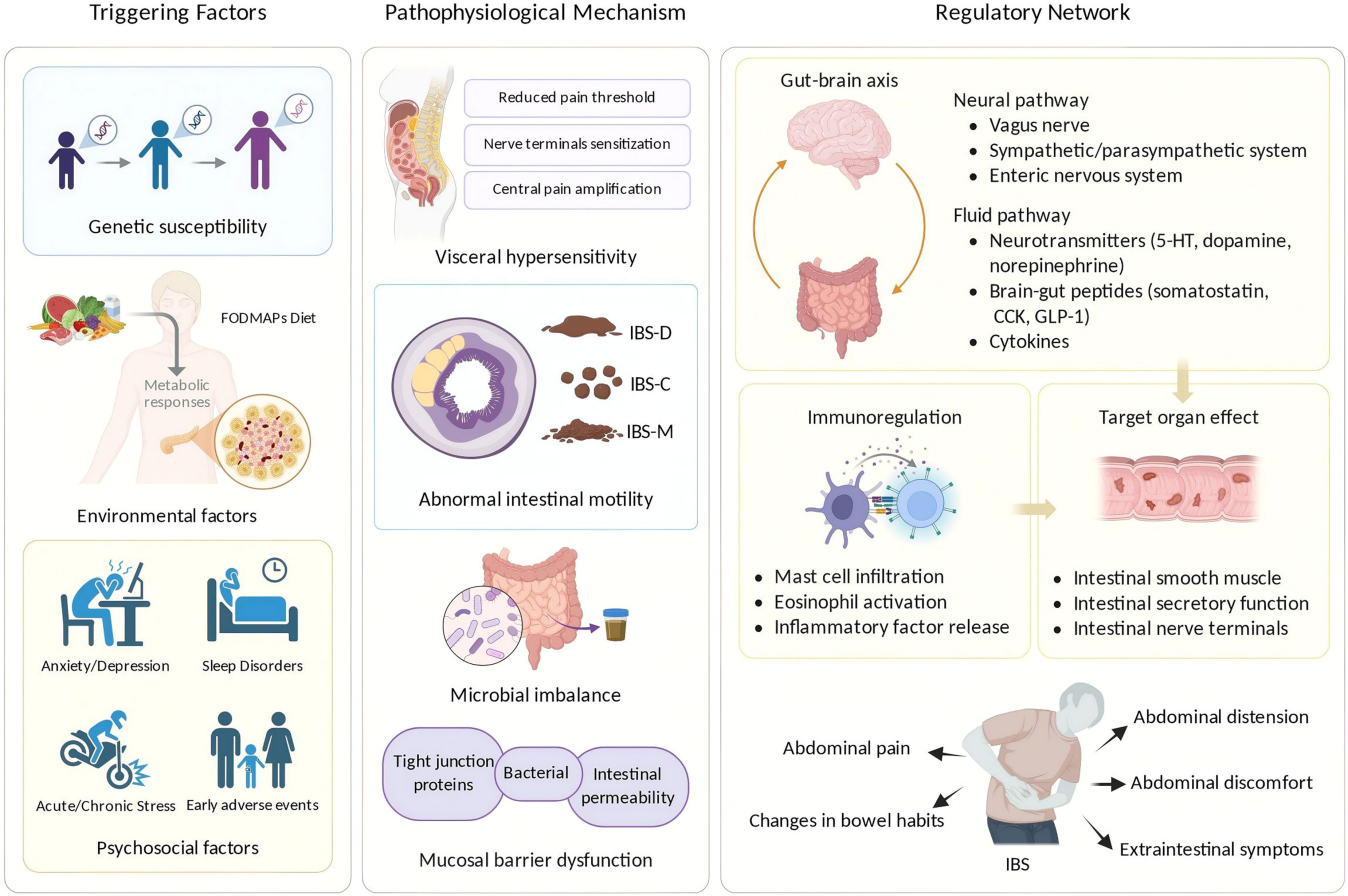
Model of irritable bowel syndrome pathogenesis, emphasizing brain-gut efferent pathways (e.g., HPA axis, autonomic nervous system) and gut-to-brain afferent pathways (e.g., vagal innervation and enteroendocrine signaling). IBS has a multifactorial pathophysiology and multiple interrelated pathways can influence the manifestation of symptoms. External factors are dominant, but internal factors such as gut microbiome, gastrointestinal immune system and genetic makeup is also likely to be crucial for the development and progression of symptoms. Food and microbial metabolism stimulate the gut’s endocrine cells to release hormones and neurotransmitters, leading to visceral pain and reducing gastrointestinal motility. Bacterial metabolites including short-chain fatty acids modify gut barrier integrity, and modulate brain and behavior via the vagus and/or directly acting brain through circulation, thereby possibly causing psychiatric comorbidities. IBS, irritable bowel syndrome; IBS-M, mixed IBS; IBS-C, constipation predominant IBS; IBS-D, diarrhea predominant IBS; FODMAPs, fermentable oligosaccharides, disaccharides, monosaccharides and polyols; 5-HT, serotonin; CCK, cholecystokinin; GLP-1, glucagon-like peptide-1.

Irritable bowel syndrome (IBS) is a common functional gastrointestinal (GI) disorder, with a global prevalence of 5%–20% (Aguilera-Lizarraga et al., 2022; Sperber et al., 2021). Typical manifestations include a history of excessive stress or irregular bowel habits, often leading to constipation and/or diarrhea, accompanied by abdominal discomfort or pain, without detectable structural abnormalities (Ashwin Dhanrajji et al., 2023; Figure 1). Epidemiological data show significant gender and geographical disparities worldwide (JohnBritto et al., 2024). As a typical disorder of brain-gut interactions, IBS frequently co-occurs with anxiety and depression, imposing substantial burdens on families, healthcare systems, and socioeconomic development (Mayer et al., 2023; Sulaimi et al., 2025). Clinical presentation is highly heterogeneous, allowing classification into diarrhea-predominant (IBS-D), constipation-predominant (IBS-C), and mixed subtypes (Enck et al., 2016; Palsson et al., 2020). Diagnosis remains symptom-based, lacking reliable biological markers (Drossman, 2006). This heterogeneity and diagnostic limitation pose significant challenges for IBS treatment.

The autonomic nervous system (ANS) exerts fine-tuned regulation over GI functions, -motility, secretion, sensation, storage, and excretion-via the enteric nervous system (ENS) (Camilleri, 2021). ANS dysfunction produces a broad symptom spectrum (ranging from diarrhea to constipation) and reduces single-target therapy efficacy through multi-organ effects, central regulatory interactions, and inter-individual differences (Mazor et al., 2025). As a unique component, the ENS closely resembles the brain in cellular structure, chemical signaling, and functional autonomy (Young, 2017). Located within the GI tract’s muscular walls, enteric neurons continuously assess luminal physical and chemical states. These neurons orchestrate muscle contractions, fluid balance, secretions, and blood circulation through circuits including interneurons, viscerofugal pathways (Hibberd et al., 2012), and motor neurons (Abdullah et al., 2020; Furness et al., 2014). Sympathetic nervous system (SNS) dysfunction is associated with mucosal immune alterations in IBS (Duan et al., 2026), while ENS neuroplastic changes may underlie persistent GI motility abnormalities in post-infectious IBS (Seguella et al., 2026). The understanding of IBS pathophysiology has shifted from early GI motility disorders to a multi-system integrated model involving vagal modulation, neuro-immune crosstalk, and the microbiota-gut-brain axis (Mayer et al., 2023). The gut microbiota (GM) influences autonomic function through neuroactive metabolites (Collins, 2014), and the ENS serotonin (5-HT) system contributes to visceral hypersensitivity (Li et al., 2024). This multi-level bidirectional network constitutes the core framework of IBS pathophysiology.

This review aims to elucidate the role of ANS dysfunction in IBS pathophysiology, providing a theoretical foundation for novel neural regulation-based therapies.

## Neuroanatomical basis of the autonomic nerve

The ANS spans both central and peripheral nervous systems. The central autonomic network comprises interconnected forebrain, brainstem, and spinal cord regions (Loewy, 1990) that integrate visceral sensation with emotional and goal-directed autonomic responses (Shields, 1993). Higher-order CNS centers (hypothalamus, amygdala, insula, anterior cingulate cortex) modulate autonomic output to the gut, mediating stress-induced GI dysfunction and emotional arousal (Barazanji et al., 2026; Browning et al., 2026). Positive and negative feedback loops regulate sympathetic and parasympathetic outputs to peripheral organs, including the immune system, and connect to central ANS output (Bonaz and Bernstein, 2013). Peripheral SNS originates from preganglionic fibers in the thoracolumbar spinal cord (T1–L3), specifically the intermediolateral gray column; parasympathetic regulation arises from brainstem nuclei and sacral segments (Johnson, 2019).

### Sympathetic nervous system

**FIGURE 2:**
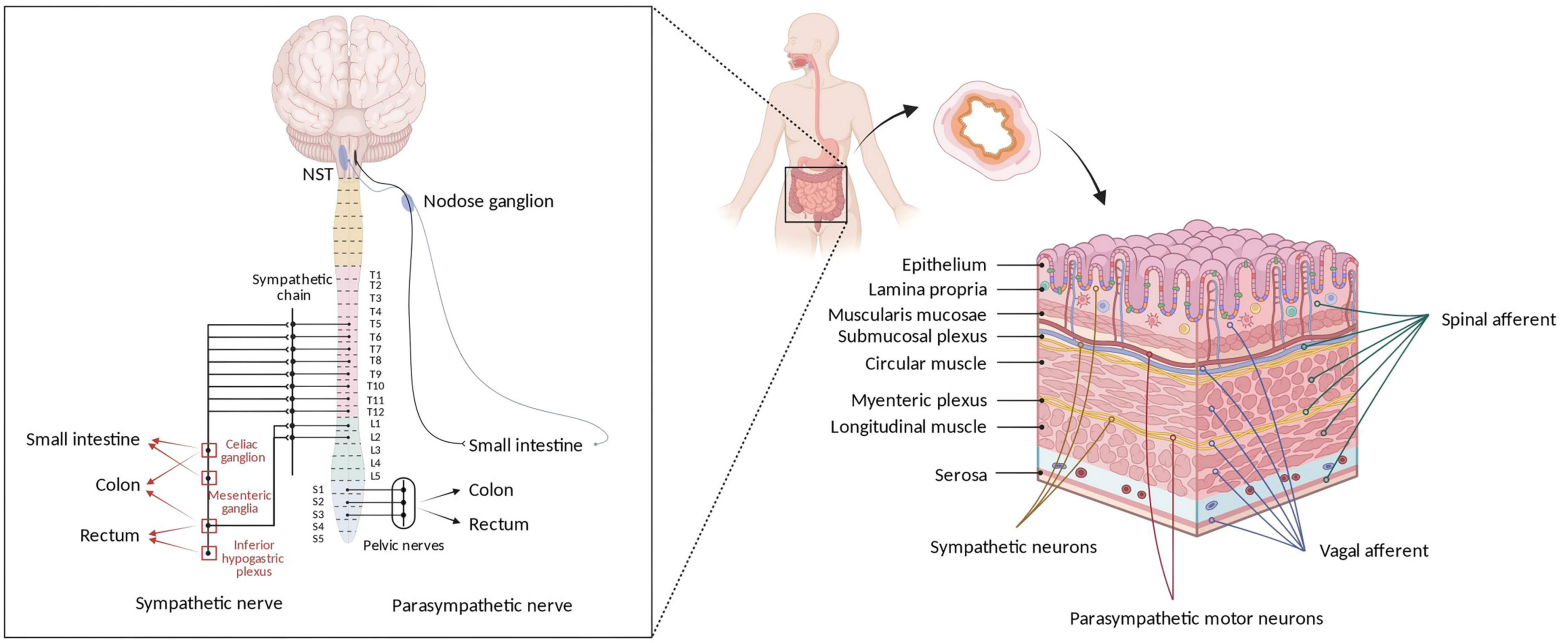
Diagram of the ANS anatomy and mode of action. The gut is innervated by extrinsic and intrinsic neurons. Extrinsic afferent neurons of the GI tract send information to the CNS through vagal or spinal afferents. Extrinsic efferent neurons, including sympathetic and parasympathetic motor neurons, project to the gut wall. Intrinsic or enteric neurons are present within the myenteric and submucosa plexus of the gut and form a complex network involved in motility, secretion and vasodilatation. The vagus nerve contains several distinct types of afferent terminals. Intraganglionic laminar endings, situated within the myenteric plexus, function as tension receptors. Intramuscular afferents, located in the muscle layer, act as stretch or length receptors. Vagal villus and crypt afferents terminate near the villus tip adjacent to the epithelium or surround the luminal ends of intestinal crypts, where they serve as chemoreceptors. The efferent vagus nerve projects into the myenteric plexus and releases neurotransmitters to regulate smooth muscle contraction and gastrointestinal motility. CNS, central nervous system; NTS, nucleus of the solitary tract; DMN, dorsal motor nucleus.

The SNS consists of preganglionic and postganglionic neurons connected in series. In the GI tract, these neurons reside in paravertebral and prevertebral ganglia-celiac, superior mesenteric, and inferior mesenteric ganglia (Figure 2; Szurszewski and Linden, 2012). They regulate motility, fluid secretion, and hormone release (Furness, 2006; Masliukov et al., 2023). Sympathetic neurons also innervate GI supply arteries and intramural arterioles (Lomax et al., 2010; Vanner and Surprenant, 1996). Sympathetic nerve fibers terminate in vessel walls, enteric plexuses (modulating vascular tone and secretomotor neurons), and perivascular regions of submucosa and mucosa (Furness, 1970). Postganglionic sympathetic neurons also interact with GI-associated lymphoid tissues and immune cells (Cervi et al., 2014). The principal neurotransmitter is norepinephrine, which acts on adrenergic receptors to inhibit enteric cholinergic neurons and smooth muscle, reducing motility and secretion (Browning et al., 2026; Wan et al., 2025).

### Parasympathetic nervous system

The vagus nerve (VN) is the main parasympathetic regulator of the GI system, conveying information on luminal osmolarity, carbohydrates, mucosal mechanical changes, and bacterial products to the CNS (Bonaz and Bernstein, 2013).

Approximately 80% of vagal fibers are afferent (transmitting taste, visceral, and somatic signals), while the remaining 20% are efferent and regulate GI motility, secretion, cardiac parasympathetic tone (Crick et al., 1994), and the cholinergic anti-inflammatory pathway (Pavlov et al., 2003). Afferent fibers arise from free terminals in the gut wall and project viscerotopically to the nucleus tractus solitarius (NTS) (Powley et al., 2019), monitoring gastric volume and intestinal nutrients while relaying non-painful sensations (nausea, satiety) (Berthoud and Neuhuber, 2000). Efferent fibers originate in the dorsal motor nucleus of the vagus and modulate smooth muscle and secretomotor effectors via cholinergic innervation of postganglionic neurons (Bonaz, 2024; Furness et al., 2014). The dorsal motor nucleus of the vagus, NTS and area postrema form the dorsal vagal complex, a primary ANS reflex center (Bonaz et al., 2017). Parasympathetic efferent signaling is primarily cholinergic (acetylcholine, nicotinic/muscarinic receptors), promoting GI motility and secretion (Layunta et al., 2022; Petsakou et al., 2023).

### Enteric nervous system

The ENS consists of the myenteric (Auerbach’s) and submucosal (Meissner’s) plexuses, forming a dense neuron-glia network capable of independently regulating gut functions (Linden and Sharkey, 2025). High-resolution imaging has identified myenteric ganglionic connectivity as the neural substrate for patterned colonic motility, such as peristalsis (Nestor-Kalinoski et al., 2022). Both sympathetic and parasympathetic inputs converge onto enteric neurons, enabling central modulation of peripheral gut functions. For example, sympathetic input inhibits mucosal secretion and regulates sphincter tone, while vagal input exerts region-and frequency-dependent effects on colonic myenteric neurons (Jiang et al., 2024; Yuki et al., 2026). Intrinsic enteric reflex circuits (e.g., peristaltic, secretory) work without central input but are tonically modulated by the autonomic system. Substance P and vasoactive intestinal peptide are key neuropeptide cotransmitters that regulate motility and secretion (Ghosh and Marshall, 2025; Jiang et al., 2024). Cholinergic, noradrenergic, or peptidergic signaling abnormalities can lead to visceral hypersensitivity and dysmotility, indicating that neural dysregulation plays a key role in GI disorders (Xu et al., 2024).

## Autonomic dysfunction in IBS patients

### Sympathovagal imbalance

Individuals with IBS often show elevated sympathetic activity, reflected by an increased low-frequency/high-frequency ratio, reduced vagal tone during rapid eye movement sleep and after meals, and a link to symptom severity (Elsenbruch and Orr, 2001; Tillisch et al., 2005). Excessive sympathetic activation suppresses intestinal hormone secretion and engages immune components via neuro-immune crosstalk, exacerbating chronic low-grade inflammation and visceral hypersensitivity (Ohman and Simrén, 2010; Wan et al., 2025). Mechanistically, stress triggers corticotropin-releasing factor (CRF) release, which binds to CRF-R1 receptors on mast cells, inducing degranulation and subsequent release of histamine, tryptase, TNF-α, and IL-6 (Labus et al., 2013; O’Malley et al., 2013). These inflammatory mediators in turn downregulate tight junction proteins, increasing intestinal permeability and facilitating bacterial translocation and immune activation (Bednarska et al., 2017; Matricon et al., 2012). Diminished vagal activity impairs its anti-inflammatory and pro-motility functions, disrupting gut-brain axis balance (Bosi et al., 2025; Mujagic et al., 2022). This imbalance leads to irregular GI motility (alternating diarrhea/constipation) and aberrant central pain processing (Higashiyama et al., 2023). Early-life stress or trauma may induce persistent autonomic dysregulation preceding IBS onset (Duan et al., 2026). Subsequent gut inflammation, visceral pain, and altered brain-gut-microbiome interactions may heighten sympathetic overactivity and vagal withdrawal (Katsumata et al., 2025; Li D. et al., 2025; Najjar et al., 2026).

### Heart rate variability

Heart rate variability (HRV) serves as a non-invasive indicator of autonomic nervous function. Prospective cohort studies confirm that abnormal HRV parameters can predict risk of progression in functional GI disorders (Ali and Chen, 2023). In subtype analysis, IBS-C patients show more pronounced parasympathetic reductions, whereas IBS-D patients are characterized primarily by sympathetic overactivation (Shi et al., 2021). Individualized HRV-guided training may be more effective than traditional high-intensity interval training in improving autonomic balance in IBS (Carrasco-Poyatos et al., 2024). Novel autonomic indicators such as cardiac deceleration capacity and periodic repolarization dynamics provide additional means to assess neural function in IBS (Boehmer et al., 2024). Furthermore, baroreflex sensitivity has shown utility in functional GI disorders (Liu et al., 2024).

### Autonomic symptoms and gastrointestinal symptoms

A significant correlation exists between autonomic symptoms and GI symptoms in IBS. Approximately 65% of IBS patients report concurrent autonomic symptoms, such as orthostatic hypotension and palpitations (MacDonald et al., 2023). Reduced vagal tone, together with GM dysbiosis and immune activation, constitutes the pathophysiological basis of IBS (Burns et al., 2022; Youssef et al., 2024). Visceral hypersensitivity correlates strongly with autonomic parameters and reduced parasympathetic activity is associated with abdominal pain severity scores (Li et al., 2024). Psychological factors exacerbate this correlation via the brain-gut axis, with IBS patients exhibiting severe anxiety and depression symptoms showing more pronounced sympathetic-parasympathetic imbalance (Staudacher et al., 2021). Psychological stress exacerbates symptoms, and prefrontal cortical dysfunction during social exclusion may reflect impaired top-down regulation (Xiong et al., 2026). Anxiety and depression commonly co-occur with IBS and are associated with greater symptom burden (Mayer et al., 2023). Transcutaneous auricular vagus nerve stimulation (taVNS) has been shown to improve autonomic function while relieving rectal hypersensitivity and abnormal defecation, supporting a central role for autonomic regulation in IBS symptoms generation (Liu et al., 2024).

## Autonomic regulation in the microbiota-gut-brain axis

### Gut microbiota metabolites

Gut microbiota plays a crucial regulatory role through its metabolites. Short-chain fatty acids (SCFAs), the primary products of dietary fiber fermentation, can influence host autonomic function by activating the cyclic adenosine monophosphate-protein kinase A signaling pathway (Deng et al., 2025). These microbial metabolites act on enteroendocrine cells (EECs), which regulate motility, secretion, inflammation, and mucosal defense by releasing 5-HT and gut hormones that affect food intake and autonomic reflexes (Dockray, 2013). Neuroactive substances produced by the microbiota, such as gamma-aminobutyric acid (GABA), can affect CNS function via the ENS-VN-brain axis (Zhong et al., 2023). Gut microbes regulate bile acids and branched-chain amino acids, which serve as signaling molecules in autonomic regulation (Ekwudo et al., 2025). Tryptophan metabolites (indoles, kynurenine) regulate gut barrier integrity, immune homeostasis, neurotransmission, and autonomic function (Leclercq et al., 2021; Wang et al., 2026). Conversely, autonomic dysfunction may reshape microbial composition and metabolite profiles (Peng and Sun, 2026). However, most evidence is correlational or derived from preclinical models. Dysbiosis-induced mast cell activation and cytokine release have been implicated in visceral hypersensitivity and autonomic imbalance (Morreale et al., 2022), whereas certain taxa may promote anti-inflammatory effects (De Luca et al., 2025). Nevertheless, the causal role of specific metabolites in IBS pathophysiology remains uncertain, and their interactions with neural, immune, and microbial factors likely vary across individuals and subtypes.

### Vagus nerve in microbiota-brain signaling

**FIGURE 3:**
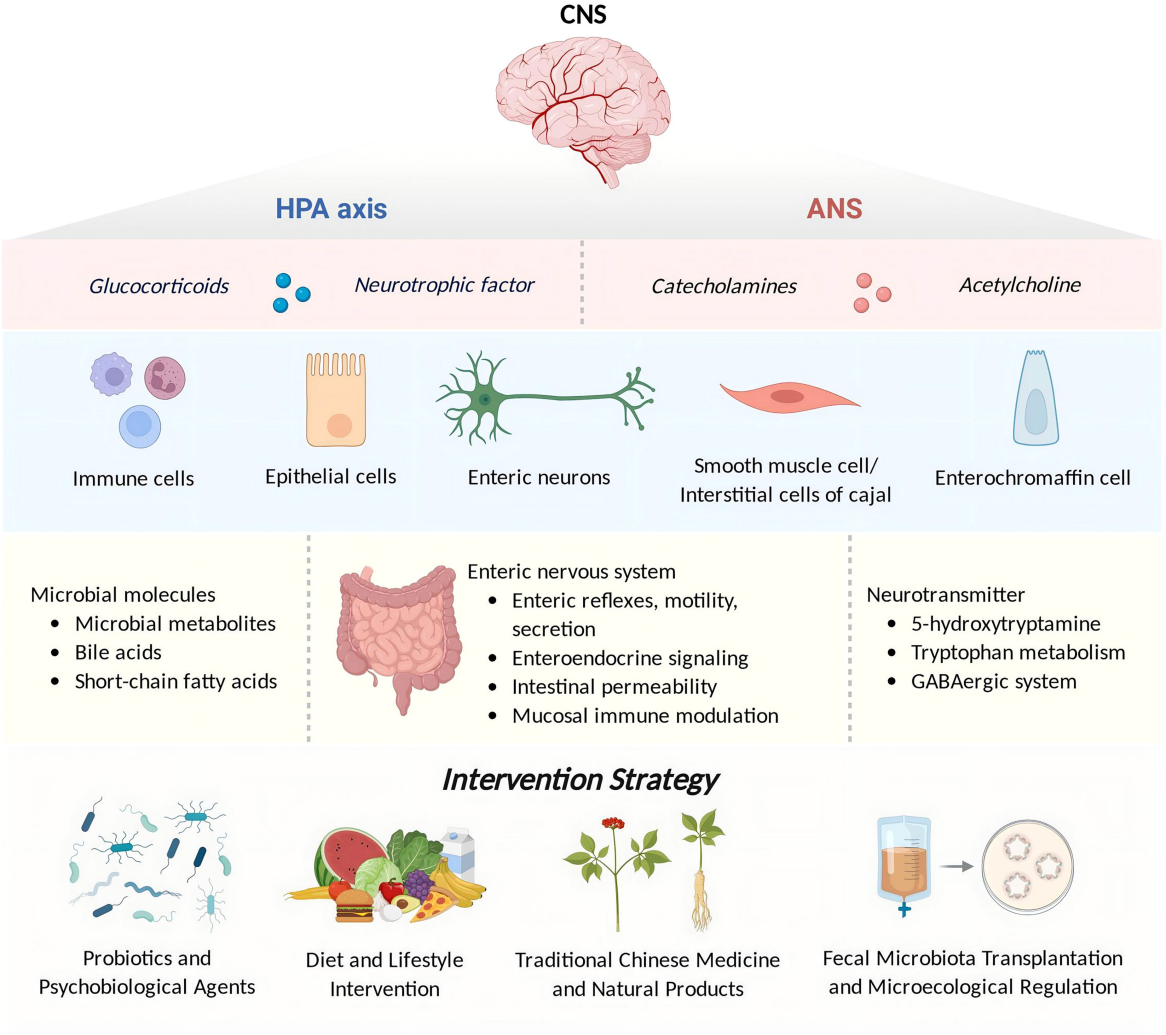
The vagus nerve provides a critical communication pathway between the gut and the brain. This gut-brain axis integrates the enteric nervous system, central nervous system, peripheral gut wall, and the hypothalamic-pituitary-adrenal (HPA) axis. Dysregulation within this network promotes visceral hypersensitivity, abnormal intestinal motility, mucosal immune activation, and alterations in the gut microbiota. Gut microbiota can activate the vagus nerve, which then relays signals from the gastrointestinal tract to the nucleus tractus solitarius (NTS). From the NTS, information is propagated through diverse neuronal pathways of the central autonomic network to produce specific physiological effects. ANS, autonomic nervous system; ENS, enteric nervous system; HPA, hypothalamo-pituitary-adrenal; PFC, prefrontal cortex; vmPFC, ventromedial prefrontal cortex; ACTH, adrenocorticotropic hormone; CRH, corticotropin-releasing hormone.

The VN is the principal neural pathway for gut-brain axis communication (Figure 3). Vagal afferents express diverse receptors, detect specific luminal stimuli, and relay signals to the brain (Sun et al., 2020), generating adaptive or maladaptive responses. Normally, GM directly activates vagal afferents only if intestinal permeability increases, as during inflammation or stress. Luminal bacteria can indirectly stimulate afferents by inducing neuroactive mediator release from EECs or gut-associated lymphoid tissue (Browning et al., 2017). Furthermore, EECs detect bacterial metabolites and subsequently activate the VN, modulating host metabolism and feeding behavior (Cummings et al., 1987; Koh et al., 2016). Beyond this cellular relay, the VN can perceive microbiota-derived signals through more direct mechanisms (Bonaz et al., 2018). For instance, microbial SCFAs activate vagal afferent fibers via pathways depending on the specific compound (Lal et al., 2001). GM changes require an intact VN, highlighting vagal afferents as essential for gut-brain regulation (Siopi et al., 2023). The VN also conveys immune signals and microbial metabolites to the brain, thereby influencing autonomic output.

### Microbiome imbalance

Dysbiosis can alter CNS and ENS functions, affecting brain-gut interactions via metabolic, immune, neural, and endocrine mechanisms (Singh et al., 2022). Colonizing germ-free animals with microbiota from IBS patients induces visceral hypersensitivity (Crouzet et al., 2013), impair intestinal permeability and alter GI transit time (De Palma et al., 2014). The development of IBS in a subgroup following acute bacterial gastroenteritis supports a role for microbiological factors in pathogenesis (Spiller, 2003). Disease severity in IBS correlates with a specific fecal microbiota profile of reduced richness and diversity (Tap et al., 2017). Additionally, antibiotic-induced dysbiosis causes persistent intestinal motility disorders through functional changes in enteric glial cells (EGCs) (Bosi et al., 2025). Psychosocial stress alters gut microbiota composition (Sandrini et al., 2015), which may contribute to symptoms in stress-reactive IBS patients (Margolis et al., 2021). Probiotics, prebiotics, and fecal microbiota transplantation have been reported to improve stress-related visceral hypersensitivity, vagal tone, or abdominal pain, though outcomes vary (Anand et al., 2022; Liu et al., 2026; Morreale et al., 2022).

### Intestinal motility disorder

Impaired intestinal transit, resulting from disrupted migrating motor complexes under parasympathetic control, promotes small intestine bacterial colonization (Van Felius et al., 2003). Conversely, accelerated transit with frequent giant migrating contractions occurs in certain diarrheal disorders like IBS-D (Chey et al., 2001). These transit alterations influence GM composition and spatial organization along the GI tract, modulated by autonomic nerves. The ANS helps shape the intestinal mucus layer by regulating mucus secretion, which in turn affects the microbiota that reside there (Macfarlane and Dillon, 2007). Aberrant expression of transient receptor potential vanilloid type-1 (TRPV1) and TRPM8 in intestinal sensory neurons contributes not only to visceral hypersensitivity but also to impaired peristalsis (Fang et al., 2024). Altered mechanical sensitivity of colonic afferent nerves, closely linked to abnormal autonomic innervation, underlies abnormal responses to visceral stimuli (Xie et al., 2023).

## Neuro-immune interaction

**FIGURE 4:**
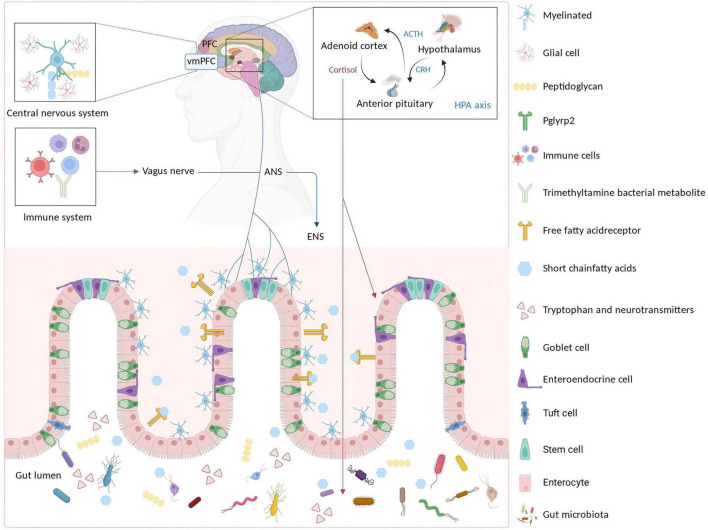
Communication between the gut and brain occurs via the endocrine system and the vagus nerve. The hypothalamic-pituitary-adrenal (HPA) axis regulates intestinal motility, gut microbiome composition, intestinal permeability, and immune activation through cortisol secretion. Through acetylcholine release and direct connections, the vagus nerve modulates immune function, intestinal permeability, and motility, often in concert with the enteric nervous system (ENS). Microbial metabolites, including short-chain fatty acids (SCFAs), neurotransmitters, and bile acids, directly influence peripheral and central nervous system activity. The microbiota also exerts its effects by modulating enteroendocrine cells, such as enterochromaffin cells. CNS, central nervous system; HPA, hypothalamic-pituitary-adrenal; ANS, autonomic nervous system.

Bidirectional neuro-immune interactions play a critical role in the IBS pathophysiology (Figure 4). Alterations in intestinal mucosal immunity are characteristic of IBS and are associated with SNS dysfunction induced by early-life stress (Duan et al., 2026). Mucosal immune cell activation is elevated, with increased numbers of T cells and mast cells (Vanuytsel et al., 2023). Key pro-inflammatory mediators in ANS-immune crosstalk in IBS are TNF-α, IL-1β, IL-6, and IL-8 (Yuan et al., 2023). Released mainly from activated monocytes or macrophages, these cytokines act on vagal afferents and enteric neurons (Schneider et al., 2023; Youssef et al., 2024). Low-level inflammation triggers nearby enteric neurons to release neuropeptides, which then activate immune cells to generate immunomodulators, affecting gut- brain communication (Yoshimoto et al., 2022). The ANS influences gut epithelial mechanisms that activate the immune system, either by directly modulating immune cell responses to bacteria or indirectly by altering bacterial access to immune cells (Alonso et al., 2008). Research indicates that sympathetic signaling influences gene transcription in gut-resident macrophages and innate lymphoid cells (Gabanyi et al., 2016; Matheis et al., 2020). Mast cell activation drives visceral hypersensitivity and pain via histamine, tryptase, and cytokines (Decraecker et al., 2025; Hussein et al., 2024), while also impairing motility through effects on ENS and smooth muscle (Hou et al., 2026). Macrophages interacting with mast cells may amplify local inflammation and pain signaling (Holter et al., 2025; Kovacs et al., 2025). Vagal activation can reverse stress-induced microbiome and immune changes (Chang H. et al., 2024), whereas vagal afferent dysregulation promotes enteric neuronal loss and barrier disruption via IL-6-mediated adrenergic signaling (Matheis et al., 2020; Ohman and Simrén, 2010). Enteric glial cells, through S100β and GDNF, modulate barrier integrity, neuronal survival, and synaptic plasticity (Gonzales and Gulbransen, 2025; Hörner et al., 2025; Schneider et al., 2024).

## Stress

Chronic or persistent stress is associated with IBS onset and exacerbation in animal and human studies, and serves as an independent predictor for post-infection IBS (Chang, 2011). IBS patients exhibit stress-induced alterations in GI motility, rectal sensitivity, autonomic tone, and hypothalamic-pituitary-adrenal HPA axis responses (Chang, 2011; Murray et al., 2004). Psychiatric comorbidities such as anxiety, depression, and hypervigilance frequently amplify stress responses, lowering the threshold for visceral perception and perpetuating autonomic dysregulation (Mazor et al., 2021; Verdonk et al., 2025). Acute and chronic stress promote cortisol release via the HPA axis and activate mast cell–nerve interactions in the intestinal mucosa, leading to release of histamine, proteases, and 5-HT, which directly stimulate enteric neurons (Buhner et al., 2009). HPA axis hyperactivity may drive sympathetic overactivation and vagal reduction via central autonomic centers (e.g., amygdala, locus coeruleus), contributing to sympathovagal imbalance (Li et al., 2026; Tse et al., 2023). Stress also compromises gut epithelial integrity and alters motility, secretions, and mucin production, promoting changes in microbial composition or activity (Collins and Bercik, 2009). Furthermore, stress-induced perturbations of the GM activate the VN and induce rapid deficits in 5-HT and dopamine neurotransmission in the brainstem and hippocampus, alongside neuroinflammation and impairments adult hippocampal neurogenesis (Siopi et al., 2023). Stress-altered gut physiology and microbiota feed back into stress responses via the brain-gut axis, reinforcing a cycle of autonomic imbalance, immune activation, and symptom exacerbation. Early adverse life events (EALs) represent a confounding factor potentially more prevalent in IBS (Chang, 2011). Videlock et al. (2009) observed that both IBS patients and controls with EALs exhibited a heightened cortisol response to visceral stress relative to those without EALs. This diminished resilience often contributes to accelerated colonic motility dysfunction (Stengel and Taché, 2009). HRV biofeedback enhances parasympathetic activity and restores sympathovagal balance (Goutham et al., 2026), while mindfulness and emotion regulation practices may improve HRV and reduce stress reactivity (Bahlinger et al., 2022; Li Z. et al., 2025). These non-pharmacological strategies collectively act on stress-ANS pathways to alleviate symptoms.

## Psychosomatic comorbidity

A systematic review found the prevalence of anxiety and depressive symptoms in IBS to be 39.1% and 28.8%, respectively (Zamani et al., 2019). The co-occurrence with mental illness is associated with more severe and disabling GI symptoms (Lee et al., 2009). Iypervigilance, catastrophizing and somatization complaints interact with ANS dysregulation, lowering sympathetic activation thresholds and hindering vagal recovery, thereby sustaining GI symptom burden (Eijsbouts et al., 2021; Katsumata et al., 2025). Reduced gray matter density in the anterior/medial thalamus of IBS patients may be linked to subclinical anxiety or depression (Davis et al., 2008). Depression can stimulate the cingulate cortex within the limbic system, which is anatomically connected to autonomic and endocrine centers regulating digestive motility and secretion. Anxiety and depression affect gut motility, secretion, and immune tone via autonomic and HPA pathways, while gut symptoms worsen mood through limbic and prefrontal circuits–a bidirectional interaction likely driving the high comorbidity and symptom persistence in IBS (Guadagnoli et al., 2025; Liang et al., 2024).

The VN contributes directly to affective behavior and indirectly to enhanced nociception in rodent models of IBS, implicating it in the pathogenesis of anxiety and depression (Bravo et al., 2011; Wang et al., 2015). The ability of certain bacterial strains to reduce depression-like behaviors and alter hippocampal protein expression also depends on the VN (Bercik et al., 2011). For instance, *Lactobacillus rhamnosus* reduced stress-induced anxiety- and depression-like behaviors by modulating forebrain GABA levels via the VN (Bravo et al., 2011). Inhibiting GABAergic transmission disinhibits vagal outputs, counteracting and limiting corticotrophin-releasing factor actions during acute stress (Travagli and Anselmi, 2016). Krieger et al. (2022) further demonstrated that a chronic lesion of gut-innervating vagal afferent neurons alters anxiety and anxiety-associated gene networks in the amygdala of male rats. As a non-pharmacological intervention, taVNS improves vagal tone, acetylcholine, and microbiota-metabolite pathways, relieving IBS symptoms and comorbid mood disorders (Liu et al., 2024).

### Oxytocin

Oxytocin influences social behavior in humans and animals (Gordon et al., 2011) and exerts anxiolytic effects in rodents, such as restoring normal GI function after stress (Zheng et al., 2010). In IBS patients, oxytocin elevates thresholds for visceral perception at doses of 20 mU/min or higher, likely through an action on visceral afferents (Louvel et al., 1996). Preautonomic paraventricular nucleus neurons provide the exclusive source of oxytocinergic innervation to the dorsal vagal complex (Richard et al., 1991). Oxytocin receptors are prominently expressed on vagal sensory neurons, particularly in nodose ganglia, enabling direct modulate of peripheral signal processing (Biddinger et al., 2024; Jiang and Travagli, 2020). The colocalization of acetylcholine and oxytocin with their receptors in hypothalamus and brainstem indicates their coordinated effects (Quintana et al., 2019). In mammals, oxytocin influences acute inflammatory pain within the paraventricular nucleus, modulates spinal nociception, and manages emotional pain processing in the amygdala (Poisbeau et al., 2018).

### Serotonin

The serotonergic system plays a complex role in anxiety and depression, with specific 5-HT receptors (1A, 2B, 2C, and 4) implicated in depression (Peng et al., 2018). In IBS patients, elevated 5-HT release over-activates mesenteric sensory neurons, heightening mucosal sensitivity; these signals can then exacerbate emotional regulation and stress responses via the dorsal raphe nucleus and locus coeruleus (Braun et al., 2007). The VN serves as the principal pathway for modulating changes in 5-HT receptor expression (Cunningham et al., 2008). Enterochromaffin cells synthesize and release 5-HT, activating receptors on VN afferent fibers before transmission via synapses or neuronal cell bodies within the nodose ganglion (Hillsley et al., 1998). This ganglion modulates cardiac vagal outflow by influencing reflexes through various receptor subtypes, particularly within the NTS (Jordan, 2005). The NTS processes serotonergic signals and relays them to the dorsal raphe nucleus, locus coeruleus, hippocampus, and cortex, affecting mood, cognition, and stress responses (Hwang and Oh, 2025). Polymorphisms in the 5-HT transporter are strongly linked to IBS risk, especially IBS-D, in Caucasians and Asians with high rectal mucosal 5-HT levels (Bi et al., 2021).

## Visceral hypersensitivity

Visceral pain in IBS arises from altered central processing of gut signals (Fitzcharles et al., 2021). Peripheral afferents respond to distension, inflammation, and chemicals (Xu et al., 2023): unmyelinated C fibers drive persistent hypersensitivity (Castro et al., 2022), while myelinated Aδ fibers mediate sharp pain (Zhou and Verne, 2025). Vagal fibers can inhibit pain by activating brainstem regions such as the periaqueductal gray and rostroventral medulla, which are involved in pain modulation (Ren et al., 1991). The amygdala receives proalgesic vagal input, for instance from cholecystokinin, and relays information to centers governing descending pain modulation (Wang et al., 2015). Together with the central autonomic network, the VN regulates intestinal fluid transport, local visceral blood flow, GI motility, the generation of cardiac and respiratory rhythms, and immune functions (Bonaz et al., 2016). This broad physiological involvement may explain the diverse sensory symptoms, such as nausea and general discomfort, that often accompany visceral dysfunction. Spinal dorsal horn neuronal hyperexcitability, receptive field expansion, and altered interneuron activity underlie central sensitization after repeated gut nociception (Wallrapp and Chiu, 2024; Zhou and Verne, 2025). Supraspinally, IBS patients show heightened cortical responses in insula, anterior cingulate, and prefrontal cortex, which correlate with pain intensity and autonomic dysregulation (Chang X. et al., 2024; Paine, 2021). In IBS-D, mast cell activation and infiltration correlate with visceral pain, indicating that immune alterations contribute to visceral hypersensitivity (Hughes et al., 2014; Patel and Shackelford, 2025). Mast cells activate nociceptive afferents via histamine, tryptase, and serotonin, serving as a key neuroimmune bridge for pain (Fialho et al., 2026). Visceral pain is frequently accompanied by autonomic symptoms, including sweating and changes in blood perfusion, which partly result from the colocalization of visceral primary afferents with sympathetic and parasympathetic fibers, enabling local crosstalk (Giamberardino and Vecchiet, 1995). GM metabolites (e.g., SCFAs, lipopolysaccharide) contribute to visceral hypersensitivity via TRP channels or toll-like receptor pathways (De Palma et al., 2022; Kotani et al., 2025). Elevated sympathetic drive may promote upper GI dysmotility in IBS (Mazur et al., 2007), whereas vagal dysfunction occurs after rectal distension (Spaziani et al., 2008). These observations align with the view that the SNS can facilitate nociception and the parasympathetic system can suppress it (Pickering et al., 2003). Accordingly, modulating vagal tone, inhibiting sympathetic overactivity, and stabilizing mast cells help reduce visceral hypersensitivity (Berthoud et al., 2025; Hou et al., 2026).

## Neuroplasticity

Neuroplasticity has been linked to IBS pathophysiology (Camilleri, 2013), but whether it is a primary driver or a secondary consequence remains unclear. Reported changes include increased mucosal nerve fiber density (Dothel et al., 2015), rapid neuronal sensitization (Buhner et al., 2009), and cortical structural alterations. These may arise from persistent low-grade gut wall inflammation and immune dysfunction (Barbara et al., 2004; Hughes et al., 2013). Pro-inflammatory cytokines and chemokines drive peripheral and central neuroplasticity by modulating neuronal excitability, synaptic remodeling, and long-term potentiation of nociceptive circuits (Defaye et al., 2022). EGCs respond to inflammatory or stress signals by switching phenotype and releasing S100β and other pro-inflammatory mediators, which sensitize enteric neurons and disrupt gut barrier function (Gonzales and Gulbransen, 2025; Morales-Soto et al., 2023). Such plastic changes persist in post-infectious IBS (Brierley and Linden, 2014) and could contribute to visceral hypersensitivity, dysmotility, and disrupted brain-gut signaling (Barth and Grill, 2025; Zhou and Verne, 2025). However, the relative contribution of neuroplasticity versus other interacting mechanisms (e.g., autonomic, immune, microbial) requires further investigation.

### Mucosal mediators

Altered release of intestinal bioactive factors such as 5-HT, histamine, and mast cell tryptase modulates intrinsic and extrinsic gut nerve activity via specific receptors (Barbara et al., 2004, 2007). In IBS, changes in 5-HT signaling may involve the sensitization of TRPV-1 (Akbar et al., 2008). Vagal afferent mechanical hypersensitivity critically depends on TRPA1 sensitization through a protease-activated receptor 2-dependent mechanism (Yu et al., 2009). Akbar et al. (2008) found that IBS patients have higher mucosal innervation density and more TRPV1-positive nerve fibers than controls, and that these fibers correlate with hypersensitivity to rectal distension and heat (Chan et al., 2003). TRP channel antagonists may therefore reduce visceral pain by modulating peripheral and central sensitization in IBS (Zhou and Verne, 2025).

### Emotional disorder

Neuroplasticity is impaired in mood disorders and in stress-exposed animal models. Vagal neurocircuits exhibit considerable plasticity, as their response phenotype is modulated by both physiological and pathophysiological states (Dockray, 2014). Chronic stress diminishes synaptic plasticity and dendritic spine density, reduces dendritic length and complexity, and impairs neurogenesis (Pittenger and Duman, 2008). Reduced neuroplasticity contributes to the comorbidity of depression, anxiety, and IBS, with glucocorticoid and brain-derived neurotrophic factor levels serving as key mediators (Zheng and Tang, 2015). Rats with IBS display depression- and anxiety-like behaviors linked to decreased brain-derived neurotrophic factor expression in the hippocampus following chronic acute combined stress (Yu et al., 2015), and antidepressant treatment can increase or reverse this deficit (Pittenger and Duman, 2008).

## Intervention strategies targeting autonomic nerves

### Neuromodulation techniques

The ENS functions as an independent unit maintaining mucosal barrier homeostasis by integrating mechanical forces, chemical signals, and microbial metabolites, providing a rationale for intervention via exogenous electrical stimulation (Sun et al., 2025). Vagus nerve stimulation (VNS) delivers targeted electrical impulses to the VN to modulate neural activity and associated physiological responses (Singh et al., 2025). Clinically, VNS ameliorates IBS symptoms, reduces abdominal pain, and normalizes dysregulated interoceptive and stress-related neural networks (Liu et al., 2024). These effects appear to be mediated by enhanced vagal signaling, increased SCFAs production, and modulation of hypothalamic circuits that control appetite and energy balance (Dong et al., 2025). In rats, electrical VNS blocks serotonin-driven endocannabinoid release in the duodenum, inhibiting visceral nociceptive transmission (Feng et al., 2014). VNS also induces plasticity in the amygdala (Hachem et al., 2018), upregulates hypothalamic corticotrophin-releasing factor, and elevates plasma adrenocorticotropin hormone and corticosterone (Hosoi et al., 2000). Such changes may prime the system, modulating synaptic plasticity in response to specific sensory inputs (Borland et al., 2018). Through heterosynaptic neuromodulation, VNS facilitates long-term synaptic modifications in motor neurons within a temporal window suitable for spike-timing-dependent plasticity (He et al., 2015). Preclinical evidence suggests VNS may exploit analogous neuroplastic mechanisms (Ni et al., 2014), though further verification is needed. Emerging non-invasive VNS may benefit abdominal pain and constipation in IBS-C (Liu et al., 2024; Shi et al., 2021), with limited evidence for other neuromodulation approaches.

### Pharmacological intervention

Within the ENS, 5-HT regulates motor and sensory functions via distinct receptor subtypes. The 5-HT3 receptor mediates fast synaptic transmission in enteric neurons, while the 5-HT4 receptor facilitates neurotransmitter release in intestinal reflex pathways (Gershon, 2004). Clinically, 5-HT3 antagonists (e.g., alosetron) alleviate chemotherapy-induced nausea and IBS-D, though constipating effects limit use in IBS-C. 5-HT4 agonists (e.g., tegaserod) enhance intestinal motility to treat IBS-C (Gershon, 2004). Alosetron also inhibits stress-induced visceral hypersensitivity by acting on the beta3 adrenoceptor (beta3-AR). The beta3-AR agonist GW427353 stimulates these receptors on human enteric neurons and adipocytes, prompting somatostatin (SST) release; SST then activates SST2 receptors on enteric neurons to reduce neuronal excitability (Schemann et al., 2010). During inflammation, reduced expression of the mucosal 5-HT transporter disrupts 5-HT signaling, a mechanism linked to both IBS-D and IBS-C pathophysiology (Gershon, 2004). Thus, precise targeting of specific 5-HT and adrenoceptor subtypes can correct GI motor and sensory dysfunction.

### Microecological intervention

Bidirectional GM-gut-brain signaling presents a novel avenue for neuromodulation. Early-life antibiotic-induced dysbiosis in mice causes persistent impairment of intestinal neuromuscular function, characterized by reduced transit, weakened nitrergic inhibition, and diminished cholinergic contraction (Bosi et al., 2025). Patients with postinfectious IBS exhibit elevated expression of the long non-coding RNA GAS5 within colonic extracellular vesicles; this upregulates the N-methyl-D-aspartate receptor subunit NR2B by inhibiting miR-23a/b, thereby mediating visceral hypersensitivity (Zhou et al., 2025). Targeted GAS5 blockade or miR-23 precursor supplementation reverses visceral hyperalgesia in mouse models. Specific probiotic formulations can also alleviate IBS symptoms, anxiety, and depression while fostering microbiome resilience after antibiotic exposure (Mullish et al., 2024).

## Conclusion

This review indicates that autonomic nervous dysfunction is a significant pathophysiological basis of IBS, though autonomic imbalance is not universal across all patients. Patients with IBS commonly exhibit sympathovagal imbalance, characterized by decreased parasympathetic tone and relative sympathetic hyperactivity. This imbalance is closely associated with visceral hypersensitivity and GI motility abnormalities, and demonstrates subtype-specific patterns, as well as variations by sex and individual stress reactivity. Autonomic dysfunction may link to neuroplastic changes and to psychosomatic comorbidities, potentially forming a self-perpetuating loop that contributes to symptom persistence and brain-gut axis dysregulation. The ANS, together with psychosocial factors, low-grade inflammation, and GM, regulates disease progression. Strategies like VNS, dietary changes (e.g., low FODMAP), psychological treatments, and 5-HT modulators or neuromodulators may help restore autonomic balance. Stress reduction and microbiome modulation are also practical. Whether autonomic imbalance is a driver or an epiphenomenon remains debated. HRV as a biomarker suffers from high variability and poor standardization. Small β-adrenergic antagonist trials have yielded inconsistent results, questioning the therapeutic value of targeting sympathetic tone alone. Most evidence for a causal link between GM changes and autonomic dysfunction is correlational, and animal models only partly mimic human psychophysiology. Thus, autonomic regulation calls for multidimensional stratification based on genetic, neural, and microbial profiles. Future multi-omics research (neuroimaging, metabolomics, single-cell sequencing) should dissect specific autonomic pathways in brain-gut crosstalk.

## References

[B1] Abdullah N. Defaye M. Altier C. (2020). Neural control of gut homeostasis. *Am. J. Physiol. Gastrointest. Liver Physiol.* 319 G718–G732. 10.1152/ajpgi.00293.2020 33026824

[B2] Aguilera-Lizarraga J. Hussein H. Boeckxstaens G. (2022). Immune activation in irritable bowel syndrome: What is the evidence? *Nat. Rev. Immunol.* 22 674–686. 10.1038/s41577-022-00700-9 35296814

[B3] Akbar A. Yiangou Y. Facer P. Walters J. Anand P. Ghosh S. (2008). Increased capsaicin receptor TRPV1-expressing sensory fibres in irritable bowel syndrome and their correlation with abdominal pain. *Gut* 57 923–929. 10.1136/gut.2007.138982 18252749 PMC2564830

[B4] Ali M. Chen J. (2023). Roles of heart rate variability in assessing autonomic nervous system in functional gastrointestinal disorders: A systematic review. *Diagnostics* 13:293. 10.3390/diagnostics13020293 36673103 PMC9857852

[B5] Alonso C. Guilarte M. Vicario M. Ramos L. Ramadan Z. Antolín M. et al. (2008). Maladaptive intestinal epithelial responses to life stress may predispose healthy women to gut mucosal inflammation. *Gastroenterology* 135 163–172.e1. 10.1053/j.gastro.2008.03.036 18455999

[B6] Anand N. Gorantla V. Chidambaram S. (2022). The role of gut dysbiosis in the pathophysiology of neuropsychiatric disorders. *Cells* 12:54. 10.3390/cells12010054 36611848 PMC9818777

[B7] Ashwin Dhanrajji P. Paresh Manilal G. Deepak Kamlakar K. Gajanan Bhagwan B. Pravin Prakash K. (2023). Complementary and alternative medicine in patients with irritable bowel syndrome: A Pilot Study. *World J. Adv. Res. Rev.* 19 363–369. 10.3748/wjg.v20.i2.346 24574705 PMC3923011

[B8] Bahlinger K. Lincoln T. Clamor A. (2022). Recovery after stress-autonomic and subjective arousal in individuals with psychosis compared to healthy controls. *Schizophr. Bull.* 48 1373–1383. 10.1093/schbul/sbac097 35998116 PMC9673261

[B9] Barazanji N. Sjödahl J. Orell G. Evripidou M. Adjeiwaah M. Icenhour A. et al. (2026). From gut feeling to gray matter: Mapping defecation urgency in irritable bowel syndrome. *Gastroenterology* Online ahead of print. 10.1053/j.gastro.2026.01.032 41763616

[B10] Barbara G. Stanghellini V. De Giorgio R. Cremon C. Cottrell G. Santini D. et al. (2004). Activated mast cells in proximity to colonic nerves correlate with abdominal pain in irritable bowel syndrome. *Gastroenterology* 126 693–702. 10.1053/j.gastro.2003.11.055 14988823

[B11] Barbara G. Wang B. Stanghellini V. de Giorgio R. Cremon C. Di Nardo G. et al. (2007). Mast cell-dependent excitation of visceral-nociceptive sensory neurons in irritable bowel syndrome. *Gastroenterology* 132 26–37. 10.1053/j.gastro.2006.11.039 17241857

[B12] BarthB. GrillW. (2025). Burst-patterned stimulation restores colonic motility in preclinical models. *Sci. Transl. Med.* 17:eadu4615. 10.1126/scitranslmed.adu4615 41191773

[B13] Bednarska O. Walter S. Casado-Bedmar M. Ström M. Salvo-Romero E. Vicario M. et al. (2017). Vasoactive intestinal polypeptide and mast cells regulate increased passage of colonic bacteria in patients with irritable bowel syndrome. *Gastroenterology* 153 948–960.e3. 10.1053/j.gastro.2017.06.051 28711627 PMC5623149

[B14] Bercik P. Park A. Sinclair D. Khoshdel A. Lu J. Huang X. et al. (2011). The anxiolytic effect of Bifidobacterium longum NCC3001 involves vagal pathways for gut-brain communication. *Neurogastroenterol. Motil.* 23 1132–1139. 10.1111/j.1365-2982.2011.01796.x 21988661 PMC3413724

[B15] Berthoud H. Neuhuber W. (2000). Functional and chemical anatomy of the afferent vagal system. *Auton. Neurosci.* 85 1–17. 10.1016/S1566-0702(00)00215-0 11189015

[B16] Berthoud H. Münzberg H. Morrison C. Neuhuber W. (2025). Gut-brain communication: Functional anatomy of vagal afferents. *Curr. Opin. Neurobiol.* 93:103058. 10.1016/j.conb.2025.103058 40451136 PMC12353194

[B17] Bi Z. Zhang S. Meng Y. Feng Y. Wang Y. Wang E. et al. (2021). Female serotonin transporter-knockout rat: A potential model of irritable bowel syndrome. *FASEB J.* 35:e21701. 10.1096/fj.202000007RRR 34143529

[B18] Biddinger J. Elson A. Fathi P. Sweet S. Nishimori K. Ayala J. et al. (2024). AgRP neurons mediate activity-dependent development of oxytocin connectivity and autonomic regulation. *Proc. Natl. Acad. Sci. U S A.* 121:e2403810121. 10.1073/pnas.2403810121 39585985 PMC11626166

[B19] Boehmer A. Schubert T. Rothe M. Keim C. Wiedenmann L. Ruckes C. et al. (2024). Angiotensin receptor-neprilysin inhibitor is associated with improved cardiac autonomic function in heart failure. *J. Am. Heart Assoc.* 13:e033538. 10.1161/JAHA.123.033538 39082399 PMC11964052

[B20] Bonaz B. (2024). Enteric neuropathy and the vagus nerve: Therapeutic implications. *Neurogastroenterol. Motil.* 37:e14842. 10.1111/nmo.14842 38873822 PMC12287902

[B21] Bonaz B. Bernstein C. (2013). Brain-gut interactions in inflammatory bowel disease. *Gastroenterology* 144 36–49. 10.1053/j.gastro.2012.10.003 23063970

[B22] Bonaz B. Bazin T. Pellissier S. (2018). The vagus nerve at the interface of the microbiota-gut-brain axis. *Front. Neurosci.* 12:49. 10.3389/fnins.2018.00049 29467611 PMC5808284

[B23] Bonaz B. Sinniger V. Pellissier S. (2016). Vagal tone: Effects on sensitivity, motility, and inflammation. *Neurogastroenterol. Motil.* 28 455–462. 10.1111/nmo.12817 27010234

[B24] Bonaz B. Sinniger V. Pellissier S. (2017). The vagus nerve in the neuro-immune axis: Implications in the pathology of the gastrointestinal tract. *Front. Immunol.* 8:1452. 10.3389/fimmu.2017.01452 29163522 PMC5673632

[B25] Borland M. Engineer C. Vrana W. Moreno N. Engineer N. Vanneste S. et al. (2018). The interval between VNS-tone pairings determines the extent of cortical map plasticity. *Neuroscience* 369 76–86. 10.1016/j.neuroscience.2017.11.004 29129793 PMC5766390

[B26] Bosi A. Banfi D. Capó J. Ponti A. Faggin S. Moro E. et al. (2025). Sex-dependent alteration of the enteric neuromuscular function after antibiotic-induced dysbiosis in juvenile mice and effect of Lactocaseibacillus rhamnosus GG. *Biomed. Pharmacother.* 189:118263. 10.1016/j.biopha.2025.118263 40516333

[B27] Braun T. Voland P. Kunz L. Prinz C. Gratzl M. (2007). Enterochromaffin cells of the human gut: Sensors for spices and odorants. *Gastroenterology* 132 1890–1901. 10.1053/j.gastro.2007.02.036 17484882

[B28] Bravo J. Forsythe P. Chew M. Escaravage E. Savignac H. Dinan T. et al. (2011). Ingestion of Lactobacillus strain regulates emotional behavior and central GABA receptor expression in a mouse via the vagus nerve. *Proc. Natl. Acad. Sci. U S A.* 108 16050–16055. 10.1073/pnas.1102999108 21876150 PMC3179073

[B29] Brierley S. Linden D. (2014). Neuroplasticity and dysfunction after gastrointestinal inflammation. *Nat. Rev. Gastroenterol. Hepatol.* 11 611–627. 10.1038/nrgastro.2014.103 25001973

[B30] Browning K. Travagli R. Pellegrini C. (2026). Central control of gastrointestinal functions in health and disease. *Physiol. Rev.* 106 971–1020. 10.1152/physrev.00010.2025 41432726 PMC12853297

[B31] Browning K. Verheijden S. Boeckxstaens G. (2017). The vagus nerve in appetite regulation, mood, and intestinal inflammation. *Gastroenterology* 152 730–744. 10.1053/j.gastro.2016.10.046 27988382 PMC5337130

[B32] Buhner S. Li Q. Vignali S. Barbara G. De Giorgio R. Stanghellini V. et al. (2009). Activation of human enteric neurons by supernatants of colonic biopsy specimens from patients with irritable bowel syndrome. *Gastroenterology* 137 1425–1434. 10.1053/j.gastro.2009.07.005 19596012

[B33] Burns G. Talley N. Keely S. (2022). Immune responses in the irritable bowel syndromes: Time to consider the small intestine. *BMC Med.* 20:115. 10.1186/s12916-022-02301-8 35354471 PMC8969236

[B34] Camilleri M. (2013). Peripheral mechanisms in irritable bowel syndrome. *N. Engl. J. Med.* 368 578–579. 10.1056/NEJMc1214185 23388017

[B35] Camilleri M. (2021). Gastrointestinal motility disorders in neurologic disease. *J. Clin. Invest.* 131:e143771. 10.1172/JCI143771 33586685 PMC7880310

[B36] Carrasco-Poyatos M. López-Osca R. Martínez-González-Moro I. Granero-Gallegos A. (2024). HRV-guided training vs traditional HIIT training in cardiac rehabilitation: A randomized controlled trial. *Geroscience* 46 2093–2106. 10.1007/s11357-023-00951-x 37853188 PMC10828341

[B37] Castro J. Garcia-Caraballo S. Maddern J. Schober G. Lumsden A. Harrington A. et al. (2022). Olorinab (APD371), a peripherally acting, highly selective, full agonist of the cannabinoid receptor 2, reduces colitis-induced acute and chronic visceral hypersensitivity in rodents. *Pain* 163 e72–e86. 10.1097/j.pain.0000000000002314 33863856 PMC8675055

[B38] Cervi A. Lukewich M. Lomax A. (2014). Neural regulation of gastrointestinal inflammation: Role of the sympathetic nervous system. *Auton. Neurosci.* 182 83–88. 10.1016/j.autneu.2013.12.003 24412637

[B39] Chan C. Facer P. Davis J. Smith G. Egerton J. Bountra C. et al. (2003). Sensory fibres expressing capsaicin receptor TRPV1 in patients with rectal hypersensitivity and faecal urgency. *Lancet* 361 385–391. 10.1016/s0140-6736(03)12392-6 12573376

[B40] Chang H. Perkins M. Novaes L. Qian F. Zhang T. Neckel P. et al. (2024). Stress-sensitive neural circuits change the gut microbiome via duodenal glands. *Cell* 187 5393–5412.e30. 10.1016/j.cell.2024.07.019 39121857 PMC11425084

[B41] Chang L. (2011). The role of stress on physiologic responses and clinical symptoms in irritable bowel syndrome. *Gastroenterology* 140 761–765. 10.1053/j.gastro.2011.01.032 21256129 PMC3039211

[B42] Chang X. Zhang H. Chen S. (2024). Neural circuits regulating visceral pain. *Commun. Biol.* 7:457. 10.1038/s42003-024-06148-y 38615103 PMC11016080

[B43] Chey W. Jin H. Lee M. Sun S. Lee K. (2001). Colonic motility abnormality in patients with irritable bowel syndrome exhibiting abdominal pain and diarrhea. *Am. J. Gastroenterol.* 96 1499–1506. 10.1111/j.1572-0241.2001.03804.x 11374689

[B44] Collins S. M. A. (2014). role for the gut microbiota in IBS. *Nat. Rev. Gastroenterol. Hepatol.* 11 497–505. 10.1038/nrgastro.2014.40 24751910

[B45] Collins S. Bercik P. (2009). The relationship between intestinal microbiota and the central nervous system in normal gastrointestinal function and disease. *Gastroenterology* 136 2003–2014. 10.1053/j.gastro.2009.01.075 19457424

[B46] Crick S. Wharton J. Sheppard M. Royston D. Yacoub M. Anderson R. et al. (1994). Innervation of the human cardiac conduction system. A quantitative immunohistochemical and histochemical study. *Circulation* 89 1697–1708. 10.1161/01.cir.89.4.1697 7908612

[B47] Crouzet L. Gaultier E. Del’Homme C. Cartier C. Delmas E. Dapoigny M. et al. (2013). The hypersensitivity to colonic distension of IBS patients can be transferred to rats through their fecal microbiota. *Neurogastroenterol. Motil.* 25 e272–e282. 10.1111/nmo.12103 23433203

[B48] Cummings J. Pomare E. Branch W. Naylor C. Macfarlane G. (1987). Short chain fatty acids in human large intestine, portal, hepatic and venous blood. *Gut* 28 1221–1227. 10.1136/gut.28.10.1221 3678950 PMC1433442

[B49] Cunningham J. Mifflin S. Gould G. Frazer A. (2008). Induction of c-Fos and DeltaFosB immunoreactivity in rat brain by Vagal nerve stimulation. *Neuropsychopharmacology* 33 1884–1895. 10.1038/sj.npp.1301570 17957222

[B50] Davis K. Pope G. Chen J. Kwan C. Crawley A. Diamant N. (2008). Cortical thinning in IBS: Implications for homeostatic, attention, and pain processing. *Neurology* 70 153–154. 10.1212/01.wnl.0000295509.30630.10 17959767

[B51] De Luca R. Arrè V. Nardone S. Incerpi S. Giannelli G. Trivedi P. et al. (2025). Gastrointestinal microbiota and inflammasomes interplay in health and disease: A gut feeling. *Gut* 75 161–175. 10.1136/gutjnl-2025-334938 40441864 PMC12703328

[B52] De Palma G. Lynch M. Lu J. et al. (2014). The adoptive transfer of anxiety and gut dysfunction from IBS patients to axenic mice through microbiota transplantation. *Gastroenterology* 146 S845–S. 10.1016/S0016-5085(14)63073-0

[B53] De Palma G. Shimbori C. Reed D. Yu Y. Rabbia V. Lu J. et al. (2022). Histamine production by the gut microbiota induces visceral hyperalgesia through histamine 4 receptor signaling in mice. *Sci. Transl. Med.* 14:eabj1895. 10.1126/scitranslmed.abj1895 35895832

[B54] Decraecker L. Cuende Estévez M. Van Remoortel S. Quan R. Stakenborg N. Wang Z. et al. (2025). Characterisation of MRGPRX2+ mast cells in irritable bowel syndrome. *Gut* 74 1068–1077. 10.1136/gutjnl-2024-334037 39988359

[B55] Defaye M. Abdullah N. Iftinca M. Hassan A. Agosti F. Zhang Z. et al. (2022). Gut-innervating TRPV1+ neurons drive chronic visceral pain via microglial P2Y12 receptor. *Cell. Mol. Gastroenterol. Hepatol.* 13 977–999. 10.1016/j.jcmgh.2021.12.012 34954381 PMC8867057

[B56] Deng F. Yang D. Qing L. Chen Y. Zou J. Jia M. et al. (2025). Exploring the interaction between the gut microbiota and cyclic adenosine monophosphate-protein kinase A signaling pathway: a potential therapeutic approach for neurodegenerative diseases. *Neural Regen. Res.* 20 3095–3112. 10.4103/NRR.NRR-D-24-00607 39589173 PMC11881707

[B57] Dockray G. (2013). Enteroendocrine cell signalling via the vagus nerve. *Curr. Opin. Pharmacol.* 13 954–958. 10.1016/j.coph.2013.09.007 24064396

[B58] Dockray G. (2014). Gastrointestinal hormones and the dialogue between gut and brain. *J. Physiol.* 592 2927–2941. 10.1113/jphysiol.2014.270850 24566540 PMC4214649

[B59] Dong T. Jann K. Wang D. Church A. (2025). Understanding whole person systems in brain-gut-microbiome research through ultra-high-field MRI imaging. *Neuroimage* 317:121360. 10.1016/j.neuroimage.2025.121360 40614884

[B60] Dothel G. Barbaro M. Boudin H. Vasina V. Cremon C. Gargano L. et al. (2015). Nerve fiber outgrowth is increased in the intestinal mucosa of patients with irritable bowel syndrome. *Gastroenterology* 148 1002–1011.e4. 10.1053/j.gastro.2015.01.042 25655556

[B61] Drossman D. (2006). The functional gastrointestinal disorders and the Rome III process. *Gastroenterology* 130 1377–1390. 10.1053/j.gastro.2006.03.008 16678553

[B62] Duan S. Kanda H. Zhu F. Okubo M. Koike T. Ohno Y. et al. (2026). Sympathetic overactivation drives colonic eosinophil infiltration linked to visceral hypersensitivity in irritable bowel syndrome. *Cell Mol Gastroenterol. Hepatol.* 20:101658. 10.1016/j.jcmgh.2025.101658 41067576 PMC12719208

[B63] Eijsbouts C. Zheng T. Kennedy N. Bonfiglio F. Anderson C. Moutsianas L. et al. (2021). Genome-wide analysis of 53,400 people with irritable bowel syndrome highlights shared genetic pathways with mood and anxiety disorders. *Nat. Genet.* 53 1543–1552. 10.1038/s41588-021-00950-8 34741163 PMC8571093

[B64] Ekwudo M. Gubert C. Hannan A. (2025). The microbiota-gut-brain axis in Huntington’s disease: Pathogenic mechanisms and therapeutic targets. *FEBS J*. 292 1282–1315. 10.1111/febs.17102 38426291 PMC11927060

[B65] Elsenbruch S. Orr W. (2001). Diarrhea- and constipation-predominant IBS patients differ in postprandial autonomic and cortisol responses. *Am. J. Gastroenterol.* 96 460–466. 10.1111/j.1572-0241.2001.03526.x 11232691

[B66] Enck P. Aziz Q. Barbara G. Farmer A. Fukudo S. Mayer E. et al. (2016). Irritable bowel syndrome. *Nat. Rev. Dis. Primers* 2:16014. 10.1038/nrdp.2016.14 27159638 PMC5001845

[B67] Fang Q. Yu L. Tian F. Chen W. Zhai Q. Zhang H. (2024). Randomized controlled trials of the effects of capsaicin or menthol on irritable bowel syndrome: A systematic review and meta-analysis. *Food Funct.* 15 11865–11874. 10.1039/d4fo04268a 39576289

[B68] Feng C. Yan X. Chen X. Wang E. Liu Q. Zhang L. et al. (2014). Vagal anandamide signaling via cannabinoid receptor 1 contributes to luminal 5-HT modulation of visceral nociception in rats. *Pain* 155 1591–1604. 10.1016/j.pain.2014.05.005 24813296

[B69] FialhoM. TonelloR. BrumE. BeckerG. BunnettN. OliveiraS. (2026). Critical role of the mast cell/tryptase/PAR2 axis in anastrozole-induced pain. *Br. J. Pharmacol.* 183 2009–2027. 10.1111/bph.70280 41391836

[B70] Fitzcharles M. Cohen S. Clauw D. Littlejohn G. Usui C. Häuser W. (2021). Nociplastic pain: Towards an understanding of prevalent pain conditions. *Lancet* 397 2098–2110. 10.1016/S0140-6736(21)00392-5 34062144

[B71] Furness J. (1970). The origin and distribution of adrenergic nerve fibres in the guinea-pig colon. *Histochemie* 21 295–306. 10.1007/BF00280899 5438338

[B72] Furness J. (2006). *The Enteric Nervous System.* Oxford: Blackwell Publishing.

[B73] Furness J. Callaghan B. Rivera L. Cho H. (2014). The enteric nervous system and gastrointestinal innervation: Integrated local and central control. *Adv. Exp. Med. Biol.* 817 39–71. 10.1007/978-1-4939-0897-4_3 24997029

[B74] Gabanyi I. Muller P. Feighery L. Oliveira T. Costa-Pinto F. Mucida D. (2016). Neuro-immune interactions drive tissue programming in intestinal macrophages. *Cell* 164 378–391. 10.1016/j.cell.2015.12.023 26777404 PMC4733406

[B75] Gershon M. (2004). Review article: Serotonin receptors and transporters – roles in normal and abnormal gastrointestinal motility. *Aliment. Pharmacol. Ther.* 20 3–14. 10.1111/j.1365-2036.2004.02180.x 15521849

[B76] Ghosh B. Marshall K. (2025). PIEZO1 in the enteric nervous system keeps the gut going. *Neuron* 113 1466–1468. 10.1016/j.neuron.2025.04.032 40403702

[B77] Giamberardino M. Vecchiet L. (1995). Visceral pain, referred hyperalgesia and outcome: New concepts. *Eur. J. Anaesthesiol. Suppl.* 10 61–66. 7641646

[B78] Gonzales J. Gulbransen B. (2025). The physiology of enteric glia. *Annu. Rev. Physiol.* 87 353–380. 10.1146/annurev-physiol-022724-105016 39546562 PMC12478476

[B79] Gordon I. Martin C. Feldman R. Leckman J. (2011). Oxytocin and social motivation. *Dev. Cogn. Neurosci.* 1 471–493. 10.1016/j.dcn.2011.07.007 21984889 PMC3185363

[B80] Goutham S. Bhargav H. Holla B. Mahadevan J. Nagendra R. Jasti N. et al. (2026). Yoga for opioid withdrawal and autonomic regulation: A randomized clinical trial. *JAMA Psychiatry* 83 238–246. 10.1001/jamapsychiatry.2025.3863 41499110 PMC12780978

[B81] Guadagnoli L. Heathcote L. Van Oudenhove L. Elsenbruch S. Keefer L. (2025). The psychobiological model of disorders of gut-brain interaction: Introduction of a novel, integrated, and testable model. *Lancet Gastroenterol. Hepatol.* 10 1041–1052. 10.1016/S2468-1253(25)00205-5 40886718

[B82] Hachem L. Wong S. Ibrahim G. (2018). The vagus afferent network: Emerging role in translational connectomics. *Neurosurg. Focus* 45:E2. 10.3171/2018.6.FOCUS18216 30173606

[B83] He K. Huertas M. Hong S. Tie X. Hell J. Shouval H. et al. (2015). Distinct eligibility traces for LTP and LTD in cortical synapses. *Neuron* 88 528–538. 10.1016/j.neuron.2015.09.037 26593091 PMC4660261

[B84] Hibberd T. Zagorodnyuk V. Spencer N. Brookes S. (2012). Identification and mechanosensitivity of viscerofugal neurons. *Neuroscience* 225 118–129. 10.1016/j.neuroscience.2012.08.040 22935724

[B85] Higashiyama M. Miura S. Hokari R. (2023). Modulation by luminal factors on the functions and migration of intestinal innate immunity. *Front. Immunol.* 14:1113467. 10.3389/fimmu.2023.1113467 36860849 PMC9968923

[B86] Hillsley K. Kirkup A. Grundy D. (1998). Direct and indirect actions of 5-hydroxytryptamine on the discharge of mesenteric afferent fibres innervating the rat jejunum. *J. Physiol.* 506 551–561. 10.1111/j.1469-7793.1998.551bw.x 9490878 PMC2230728

[B87] Holter D. Zahalka S. Brösamlen J. Radhouani M. Watzenboeck M. Artner T. et al. (2025). Mast cells activated in vitro can modulate macrophage polarization and antibacterial responses. *J. Allergy Clin. Immunol.* 156 754–773. 10.1016/j.jaci.2025.02.040 40436116

[B88] Hörner M. Burkard N. Kelm M. Leist A. Selig T. Kollmann C. et al. (2025). Glial cell line derived neurotrophic factor (GDNF) induces mucosal healing via intestinal stem cell niche activation. *Cell Prolif.* 58:e13758. 10.1111/cpr.13758 39610047 PMC11839185

[B89] Hosoi T. Okuma Y. Nomura Y. (2000). Electrical stimulation of afferent vagus nerve induces IL-1beta expression in the brain and activates HPA axis. *Am. J. Physiol. Regul. Integr. Comp. Physiol.* 279 R141–R147. 10.1152/ajpregu.2000.279.1.R141 10896875

[B90] Hou S. Ning T. Liu S. Yang X. Ye H. Zhou Y. et al. (2026). *Fusobacterium nucleatum* plays a pathogenic role in a murine model of irritable bowel syndrome by modulating intestinal purine metabolism and promoting mast cell activation. *Gut Microbes* 18:2620124. 10.1080/19490976.2026.2620124 41605881 PMC12854369

[B91] Hughes P. Harrington A. Castro J. Liebregts T. Adam B. Grasby D. et al. (2013). Sensory neuro-immune interactions differ between irritable bowel syndrome subtypes. *Gut* 62 1456–1465. 10.1136/gutjnl-2011-301856 22767422

[B92] Hughes P. Moretta M. Lim A. Grasby D. Bird D. Brierley S. et al. (2014). Immune derived opioidergic inhibition of viscerosensory afferents is decreased in Irritable Bowel Syndrome patients. *Brain Behav. Immun.* 42 191–203. 10.1016/j.bbi.2014.07.001 25063707

[B93] Hussein H. Van Remoortel S. Boeckxstaens G. (2024). Irritable bowel syndrome: When food is a pain in the gut. *Immunol. Rev.* 326 102–116. 10.1111/imr.13374 39037230

[B94] Hwang Y. Oh J. (2025). Interaction of the vagus nerve and serotonin in the gut-brain axis. *Int. J. Mol. Sci.* 26:1160. 10.3390/ijms26031160 39940928 PMC11818468

[B95] Jiang L. Yang J. Gao X. Huang J. Liu Q. Fu L. (2024). In vivo imaging of vagal-induced myenteric plexus responses in gastrointestinal tract with an optical window. *Nat. Commun.* 15:8123. 10.1038/s41467-024-52397-0 39285207 PMC11405534

[B96] Jiang Y. Travagli R. (2020). Hypothalamic-vagal oxytocinergic neurocircuitry modulates gastric emptying and motility following stress. *J. Physiol.* 598 4941–4955. 10.1113/JP280023 32864736 PMC8451654

[B97] JohnBritto J. Di Ciaula A. Noto A. Cassano V. Sciacqua A. Khalil M. et al. (2024). Gender-specific insights into the irritable bowel syndrome pathophysiology. Focus on gut dysbiosis and permeability. *Eur. J. Intern. Med.* 125 10–18. 10.1016/j.ejim.2024.03.011 38467533

[B98] Johnson O. (2019). *Autonomic Nervous System: Physiology.* Amsterdam: Elsevier, 270–281.

[B99] Jordan D. (2005). Vagal control of the heart: Central serotonergic (5-HT) mechanisms. *Exp. Physiol.* 90 175–181. 10.1113/expphysiol.2004.029058 15604109

[B100] Katsumata R. Hosokawa T. Manabe N. Mori H. Wani K. Kimura M. et al. (2025). Brain activity during a public-speaking situation in virtual reality in patients with irritable bowel syndrome and functional dyspepsia. *J. Gastroenterol.* 60 561–572. 10.1007/s00535-025-02228-w 39994039

[B101] Koh A. De Vadder F. Kovatcheva-Datchary P. Bäckhed F. (2016). From dietary fiber to host physiology: Short-chain fatty acids as key bacterial metabolites. *Cell* 165 1332–1345. 10.1016/j.cell.2016.05.041 27259147

[B102] Kotani S. Mishima Y. Kishimoto K. Oka A. Oshima N. Kawashima K. et al. (2025). Association between Toll-like receptor 9 signaling defect and developing post-infectious irritable bowel syndrome. *Front. Immunol.* 16:1672117. 10.3389/fimmu.2025.1672117 41293152 PMC12642955

[B103] Kovacs D. Heger K. Giansanti P. Iuliano C. Meissner F. Mann M. et al. (2025). Mast cells modulate macrophage biology through release of prestored CSF1. *J. Allergy Clin. Immunol.* 156 1260–1276. 10.1016/j.jaci.2025.05.022 40480612

[B104] Krieger J. Asker M. van der Velden P. Börchers S. Richard J. Maric I. et al. (2022). Neural pathway for gut feelings: Vagal interoceptive feedback from the gastrointestinal tract is a critical modulator of anxiety-like behavior. *Biol. Psychiatry* 92 709–721. 10.1016/j.biopsych.2022.04.020 35965105 PMC11438499

[B105] Labus J. Hubbard C. Bueller J. Ebrat B. Tillisch K. Chen M. et al. (2013). Impaired emotional learning and involvement of the corticotropin-releasing factor signaling system in patients with irritable bowel syndrome. *Gastroenterology* 145 1253–1261.e1–3. 10.1053/j.gastro.2013.08.016 23954313 PMC4069031

[B106] Lal S. Kirkup A. Brunsden A. Thompson D. Grundy D. (2001). Vagal afferent responses to fatty acids of different chain length in the rat. *Am. J. Physiol. Gastrointest. Liver Physiol.* 281 G907–G915. 10.1152/ajpgi.2001.281.4.G907 11557510

[B107] Layunta E. Forcén R. Grasa L. (2022). TLR2 and TLR4 modulate mouse ileal motility by the interaction with muscarinic and nicotinic receptors. *Cells* 11:1791. 10.3390/cells11111791 35681486 PMC9180263

[B108] Leclercq S. Schwarz M. Delzenne N. Stärkel P. de Timary P. (2021). Alterations of kynurenine pathway in alcohol use disorder and abstinence: A link with gut microbiota, peripheral inflammation and psychological symptoms. *Transl. Psychiatry* 11:503. 10.1038/s41398-021-01610-5 34599147 PMC8486842

[B109] Lee S. Wu J. Ma Y. Tsang A. Guo W. Sung J. (2009). Irritable bowel syndrome is strongly associated with generalized anxiety disorder: A community study. *Aliment. Pharmacol. Ther.* 30 643–651. 10.1111/j.1365-2036.2009.04074.x 19552631

[B110] Li D. Du H. Qu S. Wu J. Li Y. Xu Q. et al. (2024). Thalamic nucleus reuniens glutamatergic neurons mediate colorectal visceral pain in mice via 5-HT2B receptors. *Neurosci. Bull.* 40 1421–1433. 10.1007/s12264-024-01207-0 38739251 PMC11422542

[B111] Li D. Li Y. Zhu Z. Zhang F. Zhao Q. Jiang J. et al. (2025). The paraventricular thalamus mediates visceral pain and anxiety-like behaviors via two distinct pathways. *Neuron* 113 2310–2324.e7. 10.1016/j.neuron.2025.04.019 40345185

[B112] Li Y. Pan N. Xu C. Qiao L. Wang X. Shu W. et al. (2026). Distinct subdivisions of the cingulo-insular region and their role in modulating the cardiac sympathetic-parasympathetic balance. *Circ. Arrhythm. Electrophysiol.* 19:e013875. 10.1161/CIRCEP.125.013875 41725548 PMC12994912

[B113] Li Z. Chang J. Wang C. Jin M. Cheng I. Yau S. et al. (2025). The effects of non-pharmacological interventions on vagally-mediated heart rate variability (vmHRV) in individuals with depression: A systematic review and meta-analysis. *Neurosci. Biobehav. Rev.* 176:106300. 10.1016/j.neubiorev.2025.106300 40721201

[B114] Liang C. Wei S. Ji Y. Lin J. Jiao W. Li Z. et al. (2024). The role of enteric nervous system and GDNF in depression: Conversation between the brain and the gut. *Neurosci. Biobehav. Rev.* 167:105931. 10.1016/j.neubiorev.2024.105931 39447778

[B115] Linden D. Sharkey K. (2025). The enteric nervous system. *Curr. Biol.* 35 R979–R985. 10.1016/j.cub.2025.08.020 41118742

[B116] Liu J. Lv C. Yin M. Zhu M. Wang B. Tian J. et al. (2024). Efficacy and safety of transcutaneous auricular vagus nerve stimulation in patients with constipation-predominant irritable bowel syndrome: A single-center, single-blind, randomized controlled trial. *Am. J. Gastroenterol.* 120 2139–2153. 10.14309/ajg.0000000000003257 39689011 PMC12398348

[B117] Liu Y. Tang T. Cai H. Liu Z. (2026). Bidirectional communication between the gut microbiota and the central nervous system. *Neural Regen. Res.* 21 3411–3425. 10.4103/NRR.NRR-D-25-00434 40903950 PMC13452668

[B118] Loewy A. (1990). *Anatomy of the Autonomic Nervous System: an Overview. Central Regulation of Autonomic Functions.* New York: Oxford University Press, 3–16.

[B119] Lomax A. Sharkey K. Furness J. (2010). The participation of the sympathetic innervation of the gastrointestinal tract in disease states. *Neurogastroenterol. Motil.* 22 7–18. 10.1111/j.1365-2982.2009.01381.x 19686308

[B120] Louvel D. Delvaux M. Felez A. Fioramonti J. Bueno L. Lazorthes Y. et al. (1996). Oxytocin increases thresholds of colonic visceral perception in patients with irritable bowel syndrome. *Gut* 39 741–747. 10.1136/gut.39.5.741 9014776 PMC1383401

[B121] MacDonald D. Ji Y. Adabag S. Alonso A. Chen L. Henkle B. et al. (2023). Cardiovascular autonomic function and incident chronic obstructive pulmonary disease hospitalizations in atherosclerosis risk in communities. *Ann. Am. Thorac. Soc.* 20 1435–1444. 10.1513/AnnalsATS.202211-964OC 37364277 PMC10559138

[B122] Macfarlane S. Dillon J. (2007). Microbial biofilms in the human gastrointestinal tract. *J. Appl. Microbiol.* 102 1187–1196. 10.1111/j.1365-2672.2007.03287.x 17448154

[B123] Margolis K. Cryan J. Mayer E. (2021). The microbiota-gut-brain axis: From motility to mood. *Gastroenterology* 160 1486–1501. 10.1053/j.gastro.2020.10.066 33493503 PMC8634751

[B124] Masliukov P. Emanuilov A. Budnik A. (2023). Sympathetic innervation of the development, maturity, and aging of the gastrointestinal tract. *Anat. Rec.* 306 2249–2263. 10.1002/ar.25015 35762574

[B125] Matheis F. Muller P. Graves C. Gabanyi I. Kerner Z. Costa-Borges D. et al. (2020). Adrenergic signaling in muscularis macrophages limits infection-induced neuronal loss. *Cell* 180 64–78.e16. 10.1016/j.cell.2019.12.002 31923400 PMC7271821

[B126] Matricon J. Meleine M. Gelot A. Piche T. Dapoigny M. Muller E. et al. (2012). Review article: Associations between immune activation, intestinal permeability and the irritable bowel syndrome. *Aliment. Pharmacol. Ther.* 36 1009–1031. 10.1111/apt.12080 23066886

[B127] Mayer E. Ryu H. Bhatt R. (2023). The neurobiology of irritable bowel syndrome. *Mol. Psychiatry* 28 1451–1465. 10.1038/s41380-023-01972-w 36732586 PMC10208985

[B128] Mazor Y. Leach M. Jones M. Ejova A. Fisher C. Joffe D. et al. (2025). Prospective evaluation of autonomic function and intestinal blood flow in health and irritable bowel syndrome shows differences limited to patients with constipation predominance. *Neurogastroenterol. Motil.* 37:e14975. 10.1111/nmo.14975 39627962

[B129] Mazor Y. Trieu R. Prott G. Jones M. Ejova A. Kellow J. et al. (2021). Volumetric rectal perception testing: Is it clinically relevant? Results from a large patient cohort. *Am. J. Gastroenterol.* 116 2419–2429. 10.14309/ajg.0000000000001526 34608885

[B130] Mazur M. Furgała A. Jabłoński K. Madroszkiewicz D. Ciećko-Michalska I. Bugajski A. et al. (2007). Dysfunction of the autonomic nervous system activity is responsible for gastric myoelectric disturbances in the irritable bowel syndrome patients. *J. Physiol. Pharmacol.* 58 131–139. 17901589

[B131] Morales-Soto W. Gonzales J. Jackson W. Gulbransen B. (2023). Enteric glia promote visceral hypersensitivity during inflammation through intercellular signaling with gut nociceptors. *Sci. Signal.* 16:eadg1668. 10.1126/scisignal.adg1668 37988454 PMC10733972

[B132] Morreale C. Bresesti I. Bosi A. Baj A. Giaroni C. Agosti M. et al. (2022). Microbiota and pain: Save your gut feeling. *Cells* 11:971. 10.3390/cells11060971 35326422 PMC8946251

[B133] Mujagic Z. Kasapi M. Jonkers D. Garcia-Perez I. Vork L. Weerts Z. et al. (2022). Integrated fecal microbiome-metabolome signatures reflect stress and serotonin metabolism in irritable bowel syndrome. *Gut Microbes* 14:2063016. 10.1080/19490976.2022.2063016 35446234 PMC9037519

[B134] Mullish B. Michael D. Dabcheva M. Webberley T. Coates N. John D. et al. (2024). A double-blind, randomized, placebo-controlled study assessing the impact of probiotic supplementation on the symptoms of irritable bowel syndrome in females. *Neurogastroenterol. Motil.* 36:e14751. 10.1111/nmo.14751 38287443

[B135] Murray C. Flynn J. Ratcliffe L. Jacyna M. Kamm M. Emmanuel A. (2004). Effect of acute physical and psychological stress on gut autonomic innervation in irritable bowel syndrome. *Gastroenterology* 127 1695–1703. 10.1053/j.gastro.2004.08.057 15578507

[B136] Najjar S. Kildegaard H. Talati A. Goncalves P. Del Colle A. Huang Z. et al. (2026). Enteric and sympathetic nervous system pathways mediate early life stress effects on gut motility and pain: Mechanistic findings with human correlation. *Gastroenterology* Online ahead of print. 10.1053/j.gastro.2026.02.030 41850537 PMC13004275

[B137] Nestor-Kalinoski A. Smith-Edwards K. Meerschaert K. Margiotta J. Rajwa B. Davis B. et al. (2022). Unique neural circuit connectivity of mouse proximal, middle, and distal colon defines regional colonic motor patterns. *Cell. Mol. Gastroenterol. Hepatol.* 13 309–337.e3. 10.1016/j.jcmgh.2021.08.016 34509687 PMC8703201

[B138] Ni Z. Gunraj C. Kailey P. Cash R. Chen R. (2014). Heterosynaptic modulation of motor cortical plasticity in human. *J. Neurosci.* 34 7314–7321. 10.1523/JNEUROSCI.4714-13.2014 24849363 PMC6608185

[B139] Ohman L. Simrén M. (2010). Pathogenesis of IBS: Role of inflammation, immunity and neuroimmune interactions. *Nat. Rev. Gastroenterol. Hepatol.* 7 163–173. 10.1038/nrgastro.2010.4 20101257

[B140] O’Malley D. Cryan J. Dinan T. (2013). Crosstalk between interleukin-6 and corticotropin-releasing factor modulate submucosal plexus activity and colonic secretion. *Brain Behav. Immun.* 30 115–124. 10.1016/j.bbi.2013.01.078 23369733

[B141] Paine P. (2021). Review article: Current and future treatment approaches for pain in IBS. *Aliment. Pharmacol. Ther.* 54 S75–S88. 10.1111/apt.16550 34927753

[B142] Palsson O. Whitehead W. Törnblom H. Sperber A. Simren M. (2020). Prevalence of Rome IV functional bowel disorders among adults in the United States, Canada, and the United Kingdom. *Gastroenterology* 158 1262–1273.e3. 10.1053/j.gastro.2019.12.021 31917991

[B143] Patel N. Shackelford K. B. (2025). “Irritable Bowel Syndrome,” in *StatPearls*. Treasure Island, FL: StatPearls Publishing.

[B144] Pavlov V. Wang H. Czura C. Friedman S. Tracey K. (2003). The cholinergic anti-inflammatory pathway: A missing link in neuroimmunomodulation. *Mol. Med.* 9 125–134. 14571320 PMC1430829

[B145] Peng L. Song D. Li B. Verkhratsky A. (2018). Astroglial 5-HT2B receptor in mood disorders. *Expert Rev. Neurother.* 18 435–442. 10.1080/14737175.2018.1458612 29600729

[B146] Peng M. Sun H. (2026). The indole-brain connection: Neuroimmune mechanisms and therapy. *Curr. Opin. Immunol.* 98:102708. 10.1016/j.coi.2025.102708 41385980

[B147] Petsakou A. Liu Y. Liu Y. Comjean A. Hu Y. Perrimon N. (2023). Cholinergic neurons trigger epithelial Ca2+ currents to heal the gut. *Nature* 623 122–131. 10.1038/s41586-023-06627-y 37722602 PMC10699467

[B148] Pickering A. Boscan P. Paton J. (2003). Nociception attenuates parasympathetic but not sympathetic baroreflex via NK1 receptors in the rat nucleus tractus solitarii. *J. Physiol.* 551 589–599. 10.1113/jphysiol.2003.046615 12813142 PMC2343224

[B149] Pittenger C. Duman R. (2008). Stress, depression, and neuroplasticity: A convergence of mechanisms. *Neuropsychopharmacology* 33 88–109. 10.1038/sj.npp.1301574 17851537

[B150] Poisbeau P. Grinevich V. Charlet A. (2018). Oxytocin signaling in pain: Cellular, circuit, system, and behavioral levels. *Curr. Top. Behav. Neurosci.* 35 193–211. 10.1007/7854_2017_14 28942595

[B151] Powley T. Jaffey D. McAdams J. Baronowsky E. Black D. Chesney L. et al. (2019). Vagal innervation of the stomach reassessed: Brain-gut connectome uses smart terminals. *Ann. N. Y. Acad. Sci.* 1454 14–30. 10.1111/nyas.14138 31268562 PMC6810743

[B152] Quintana D. Rokicki J. van der Meer D. Alnæs D. Kaufmann T. Córdova-Palomera A. et al. (2019). Oxytocin pathway gene networks in the human brain. *Nat. Commun.* 10:668. 10.1038/s41467-019-08503-8 30737392 PMC6368605

[B153] Ren K. Randich A. Gebhart G. (1991). Effects of electrical stimulation of vagal afferents on spinothalamic tract cells in the rat. *Pain* 44 311–319. 10.1016/0304-3959(91)90102-4 1646992

[B154] Richard P. Moos F. Freund-Mercier M. (1991). Central effects of oxytocin. *Physiol. Rev.* 71 331–370. 10.1152/physrev.1991.71.2.331 1672455

[B155] Sandrini S. Aldriwesh M. Alruways M. Freestone P. (2015). Microbial endocrinology: Host-bacteria communication within the gut microbiome. *J. Endocrinol.* 225 R21–R34. 10.1530/JOE-14-0615 25792117

[B156] Schemann M. Hafsi N. Michel K. Kober O. Wollmann J. Li Q. et al. (2010). The beta3-adrenoceptor agonist GW427353 (Solabegron) decreases excitability of human enteric neurons via release of somatostatin. *Gastroenterology* 138 266–274. 10.1053/j.gastro.2009.09.046 19786030

[B157] Schneider K. Blank N. Alvarez Y. Thum K. Lundgren P. Litichevskiy L. et al. (2023). The enteric nervous system relays psychological stress to intestinal inflammation. *Cell* 186 2823–2838.e20. 10.1016/j.cell.2023.05.001 37236193 PMC10330875

[B158] Schneider L. Schneider R. Hamza E. Wehner S. (2024). Extracellular matrix substrates differentially influence enteric glial cell homeostasis and immune reactivity. *Front. Immunol.* 15:1401751. 10.3389/fimmu.2024.1401751 39119341 PMC11306135

[B159] Seguella L. Thomasi B. Franzin S. McClain J. Lavalle R. Zilli A. et al. (2026). Hyperactive enteric glia contribute to persistent dysmotility following inflammation by driving aberrant excitatory responses in neurons. *Cell Mol. Gastroenterol. Hepatol.* 20:101634. 10.1016/j.jcmgh.2025.101634 40992736 PMC12662119

[B160] Shi X. Hu Y. Zhang B. Li W. Chen J. Liu F. (2021). Ameliorating effects and mechanisms of transcutaneous auricular vagal nerve stimulation on abdominal pain and constipation. *JCI Insight* 6:e150052. 10.1172/jci.insight.150052 34138761 PMC8410029

[B161] Shields R. (1993). Functional anatomy of the autonomic nervous system. *J. Clin. Neurophysiol.* 10 2–13. 10.1097/00004691-199301000-00002 8458993

[B162] Singh N. Singh V. Rai S. Mishra V. Vamanu E. Singh M. (2022). Deciphering the gut microbiome in neurodegenerative diseases and metagenomic approaches for characterization of gut microbes. *Biomed. Pharmacother.* 156:113958. 10.1016/j.biopha.2022.113958 36411639

[B163] Singh P. Chaudhary M. Kazmi J. Kuschner C. Volpe B. Chaudhuri T. et al. (2025). Vagus nerve stimulation: A targeted approach for reducing tissue-specific ischemic reperfusion injury. *Biomed. Pharmacother.* 184:117898. 10.1016/j.biopha.2025.117898 39923406

[B164] Siopi E. Galerne M. Rivagorda M. Saha S. Moigneu C. Moriceau S. et al. (2023). Gut microbiota changes require vagus nerve integrity to promote depressive-like behaviors in mice. *Mol. Psychiatry* 28 3002–3012. 10.1038/s41380-023-02071-6 37131071 PMC10615761

[B165] Spaziani R. Bayati A. Redmond K. Bajaj H. Mazzadi S. Bienenstock J. et al. (2008). Vagal dysfunction in irritable bowel syndrome assessed by rectal distension and baroreceptor sensitivity. *Neurogastroenterol. Motil.* 20 336–342. 10.1111/j.1365-2982.2007.01042.x 18179607

[B166] Sperber A. Bangdiwala S. Drossman D. Ghoshal U. Simren M. Tack J. et al. (2021). Worldwide prevalence and burden of functional gastrointestinal disorders, results of rome foundation global study. *Gastroenterology* 160 99–114.e3. 10.1053/j.gastro.2020.04.014 32294476

[B167] Spiller R. (2003). Postinfectious irritable bowel syndrome. *Gastroenterology* 124 1662–1671. 10.1016/s0016-5085(03)00324-x 12761724

[B168] Staudacher H. Mikocka-Walus A. Ford A. (2021). Common mental disorders in irritable bowel syndrome: Pathophysiology, management, and considerations for future randomised controlled trials. *Lancet Gastroenterol. Hepatol.* 6 401–410. 10.1016/S2468-1253(20)30363-0 33587890

[B169] Stengel A. Taché Y. (2009). Neuroendocrine control of the gut during stress: Corticotropin-releasing factor signaling pathways in the spotlight. *Annu. Rev. Physiol.* 71 219–239. 10.1146/annurev.physiol.010908.163221 18928406 PMC2714186

[B170] Sulaimi F. Ong T. Tang A. Quek J. Pillay R. Low D. et al. (2025). Risk factors for developing irritable bowel syndrome: Systematic umbrella review of reviews. *BMC Med.* 23:103. 10.1186/s12916-025-03930-5 39985070 PMC11846330

[B171] Sun L. Li J. Nie Y. (2020). Gut hormones in microbiota-gut-brain cross-talk. *Chin. Med. J.* 133 826–833. 10.1097/CM9.0000000000000706 32132364 PMC7147657

[B172] Sun N. Cao L. Xia W. Wang J. Wu Q. (2025). Gut sensory neurons as regulators of neuro-immune-microbial interactions: From molecular mechanisms to precision therapy for IBD/IBS. *J. Neuroinflammation* 22 172. 10.1186/s12974-025-03500-9 40605050 PMC12219063

[B173] Szurszewski J. Linden D. R. (2012). “Physiology of prevertebral sympathetic ganglia,” in *Physiology of the gastrointestinal tract*, eds Johnson L. R. Ghishan F. K. Kaunitz J. D. Merchant J. L. Said H. M. Wood J. D. (Amsterdam: Elsevier), 583–627. 10.1016/B978-0-12-382026-6.00020-8

[B174] Tap J. Derrien M. Törnblom H. Brazeilles R. Cools-Portier S. Doré J. et al. (2017). Identification of an intestinal microbiota signature associated With severity of irritable bowel syndrome. *Gastroenterology* 152 111–123.e8. 10.1053/j.gastro.2016.09.049 27725146

[B175] Tillisch K. Mayer E. Labus J. Stains J. Chang L. Naliboff B. (2005). Sex specific alterations in autonomic function among patients with irritable bowel syndrome. *Gut* 54 1396–1401. 10.1136/gut.2004.058685 15923667 PMC1774694

[B176] Travagli R. Anselmi L. (2016). Vagal neurocircuitry and its influence on gastric motility. *Nat. Rev. Gastroenterol. Hepatol.* 13 389–401. 10.1038/nrgastro.2016.76 27251213 PMC5605144

[B177] Tse D. Privitera L. Norton A. Gobbo F. Spooner P. Takeuchi T. et al. (2023). Cell-type-specific optogenetic stimulation of the locus coeruleus induces slow-onset potentiation and enhances everyday memory in rats. *Proc. Natl. Acad. Sci. U S A.* 120:e2307275120. 10.1073/pnas.2307275120 37931094 PMC10655220

[B178] Van Felius I. Akkermans L. Bosscha K. Verheem A. Harmsen W. Visser M. et al. (2003). Interdigestive small bowel motility and duodenal bacterial overgrowth in experimental acute pancreatitis. *Neurogastroenterol. Motil.* 15 267–276. 10.1046/j.1365-2982.2003.00410.x 12787336

[B179] Vanner S. Surprenant A. (1996). Neural reflexes controlling intestinal microcirculation. *Am. J. Physiol.* 271 G223–G230. 10.1152/ajpgi.1996.271.2.G223 8770037

[B180] Vanuytsel T. Bercik P. Boeckxstaens G. (2023). Understanding neuroimmune interactions in disorders of gut-brain interaction: From functional to immune-mediated disorders. *Gut* 72 787–798. 10.1136/gutjnl-2020-320633 36657961 PMC10086308

[B181] Verdonk C. Ajijola O. Khalsa S. (2025). Toward a multidisciplinary neurobiology of interoception and mental health. *Curr. Opin. Neurobiol.* 94:103084. 10.1016/j.conb.2025.103084 40616883

[B182] Videlock E. Adeyemo M. Licudine A. Hirano M. Ohning G. Mayer M. et al. (2009). Childhood trauma is associated with hypothalamic-pituitary-adrenal axis responsiveness in irritable bowel syndrome. *Gastroenterology* 137 1954–1962. 10.1053/j.gastro.2009.08.058 19737564 PMC2789911

[B183] Wallrapp A. Chiu I. (2024). Neuroimmune interactions in the intestine. *Annu. Rev. Immunol.* 42 489–519. 10.1146/annurev-immunol-101921-042929 38941607 PMC13058849

[B184] Wan Y. Cao C. Zeng W. (2025). The sympathetic neurons in the gut: Perspectives on metabolic and immune health and diseases. *Curr. Opin. Neurobiol.* 93:103051. 10.1016/j.conb.2025.103051 40446451

[B185] Wang E. Li W. Yan X. Chen X. Liu Q. Feng C. et al. (2015). Vagal afferent-dependent cholecystokinin modulation of visceral pain requires central amygdala NMDA-NR2B receptors in rats. *Neurogastroenterol. Motil.* 27 1333–1343. 10.1111/nmo.12633 26197883

[B186] Wang T. Luo Y. Feng W. Jiang L. Yu S. He Y. et al. (2026). Gut microbial metabolism via hippocampal indole-AhR signaling regulates emotional symptoms. *Cell Metab.* 10.1016/j.cmet.2026.03.003 [Epub ahead of print]. 41928504

[B187] Xie Z. Feng J. Hibberd T. Chen B. Zhao Y. Zang K. et al. (2023). Piezo2 channels expressed by colon-innervating TRPV1-lineage neurons mediate visceral mechanical hypersensitivity. *Neuron* 111 526–538.e4. 10.1016/j.neuron.2022.11.015 36563677 PMC9957938

[B188] Xiong N. Mi Q. Sun Q. Liu Q. Fan T. (2026). Disrupted prefrontal regulation of social rejection in irritable bowel syndrome. *Psychother. Psychosom.* 1–17. 10.1159/000551116 [Epub ahead of print]. 41746847

[B189] Xu C. Jiang C. Tian Y. Liu Y. Zhang H. Xiang Z. et al. (2024). Nervous system in colorectal cancer. *Cancer Lett.* 611:217431. 10.1016/j.canlet.2024.217431 39725147

[B190] Xu J. McGinnis A. Ji R. (2023). Piezo2 mediates visceral mechanosensation: A new therapeutic target for gut pain? *Neuron* 111 450–452. 10.1016/j.neuron.2023.01.011 36796326

[B191] Yoshimoto T. Oshima T. Huang X. Tomita T. Fukui H. Miwa H. (2022). Microinflammation in the intestinal mucosa and symptoms of irritable bowel syndrome. *J. Gastroenterol.* 57 62–69. 10.1007/s00535-021-01838-4 34854984

[B192] Young V. (2017). The role of the microbiome in human health and disease: An introduction for clinicians. *BMJ* 356:j831. 10.1136/bmj.j831 28298355

[B193] Youssef A. Rehman A. Elebasy M. Roper J. Sheikh S. Karhausen J. et al. (2024). Vagal stimulation ameliorates murine colitis by regulating SUMOylation. *Sci. Transl. Med.* 16:eadl2184. 10.1126/scitranslmed.adl2184 39565873 PMC12351663

[B194] Yu S. Gao G. Peterson B. Ouyang A. (2009). TRPA1 in mast cell activation-induced long-lasting mechanical hypersensitivity of vagal afferent C-fibers in guinea pig esophagus. *Am. J. Physiol. Gastrointest Liver Physiol.* 297 G34–G42. 10.1152/ajpgi.00068.2009 19423751

[B195] Yu Y. Wu S. Li J. Wang R. Xie X. Yu X. et al. (2015). The effect of curcumin on the brain-gut axis in rat model of irritable bowel syndrome: Involvement of 5-HT-dependent signaling. *Metab. Brain Dis.* 30 47–55. 10.1007/s11011-014-9554-z 24807589

[B196] Yuan Y. Wang X. Huang S. Wang H. Shen G. (2023). Low-level inflammation, immunity, and brain-gut axis in IBS: Unraveling the complex relationships. *Gut Microbes* 15:2263209. 10.1080/19490976.2023.2263209 37786296 PMC10549202

[B197] Yuki N. Sawamura T. Mori A. Yamaguchi H. Horii Y. Shiina T. et al. (2026). Involvement of the hypothalamus-raphe magnus-spinal defecation center axis in stress-induced defecation in rats. *Commun. Biol.* 9:411. 10.1038/s42003-026-09779-5 41922653 PMC13043784

[B198] Zamani M. Alizadeh-Tabari S. Zamani V. (2019). Systematic review with meta-analysis: The prevalence of anxiety and depression in patients with irritable bowel syndrome. *Aliment. Pharmacol. Ther.* 50 132–143. 10.1111/apt.15325 31157418

[B199] Zheng J. Babygirija R. Bülbül M. Cerjak D. Ludwig K. Takahashi T. (2010). Hypothalamic oxytocin mediates adaptation mechanism against chronic stress in rats. *Am. J. Physiol. Gastrointest. Liver Physiol.* 299 G946–G953. 10.1152/ajpgi.00483.2009 20689056 PMC2957337

[B200] Zheng Z. Tang H. (2015). Decreased neuroplasticity may play a role in irritable bowel syndrome: Implication from the comorbidity of depression and irritable bowel syndrome. *J. Neurogastroenterol. Motil.* 21 298–299. 10.5056/jnm14158 25843085 PMC4398247

[B201] Zhong H. Wang S. Zhang R. Zhuang Y. Li L. Yi S. et al. (2023). Supplementation with high-GABA-producing Lactobacillus plantarum L5 ameliorates essential tremor triggered by decreased gut bacteria-derived GABA. *Transl. Neurodegener.* 12:58. 10.1186/s40035-023-00391-9 38093327 PMC10717605

[B202] Zhou Q. Verne G. (2025). Molecular mechanisms and pathways in visceral pain. *Cells* 14:1146. 10.3390/cells14151146 40801578 PMC12345894

[B203] Zhou Q. Yang L. Verne Z. Zhang B. Fields J. Thacker A. et al. (2025). Human colonic EVs induce murine enteric neuroplasticity via the lncRNA GAS5/miR-23/NMDA NR2B axis. *JCI Insight* 10:e178631. 10.1172/jci.insight.178631 40059833 PMC11949048

